# Icariin Enhances the Enzymatic Activity of N‐acetylgalactosaminidase to Augment Akkermansia Abundance in Gut Microbiota for Improved PD‐1 Blockade Efficacy in Tumor Suppression

**DOI:** 10.1002/advs.202519942

**Published:** 2026-04-22

**Authors:** Shuangying Qiao, Liu Yang, Haibang Hao, Qiuxia Ding, Feng Ding, Zheng Chen, Jinfang Zhang, Yun He, Meng Li, Jun Xu, Chao Wang, Aiping Lu, Fangfei Li

**Affiliations:** ^1^ Shum Yiu Foon Sum Bik Chuen Memorial Centre for Cancer and Inflammation Research (CCIR) School of Chinese Medicine Hong Kong Baptist University Hong Kong SAR P. R. China; ^2^ Institute of Integrated Bioinfomedicine and Translational Science (IBTS) School of Chinese Medicine Hong Kong Baptist University Hong Kong SAR P. R. China; ^3^ Institute of Precision Medicine and Innovative Drug Discovery (PMID) School of Chinese Medicine Hong Kong Baptist University Hong Kong SAR P. R. China; ^4^ Department of Laboratory Medicine The Third Affiliated Hospital of Shenzhen University Shenzhen P. R. China; ^5^ Department of Urology The Third Affiliated Hospital of Shenzhen University Shenzhen P. R. China; ^6^ Institute of Chinese Medicine Guangdong Pharmaceutical University Guangzhou Guangdong P. R. China

**Keywords:** Akkermansia, cancer, gut microbiota, icariin, immunotherapy, tumor immune microenvironment

## Abstract

Akkermansia (Akk) is a commensal bacterium in gut microbiota known to enhance anti‐tumor immunity and improve responses to immunotherapy. However, the clinical translation of Akk‐based therapies still faces critical challenges, including intestinal colonization difficulties, inter‐patient variability, and safety risks. Here, we identified the flavonoid icariin as a novel prebiotic candidate that could specifically enrich intestinal Akk abundance under various conditions both in vivo and in vitro. Oral administration of icariin selectively increased intestinal Akk abundance in tumor‐bearing and non‐tumor mice, regardless of their immunocompetent or immunodeficient status. Moreover, in vitro assays confirmed that icariin promoted Akk growth in a mucin‐dependent manner. Transcriptomic and metabolomic analyses revealed that icariin enhanced mucin‐associated metabolic pathways to support Akk growth. Mechanistically, we found that icariin increased the enzymatic activity of N‐acetylgalactosaminidase Amuc_0920 by stabilizing key residues at the binding sites for the substrate GalNAc, which enhanced mucin catabolism and promoted Akk growth. Functionally, we further found that this Akk enrichment enhanced intratumoral CD8+ T cell functions by single‐cell RNA sequencing. Consequently, icariin‐induced Akk enrichment significantly enhanced the efficacy of anti‐PD‐1 immunotherapy in tumor mouse models. These findings establish icariin as a promising Akk‐targeting prebiotic that selectively enriches Akk to improve cancer immunotherapy.

## Graphical abstract

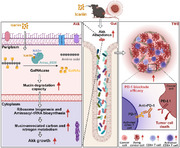

## 1 Introduction

Cancer is a systemic disease that involves complex interactions across the body [1]. Beyond the influences from the intrinsic systems such as the immune system and metabolic processes, increasing evidence reveals that the gut microbiota, a complex and diverse microbial ecosystem residing in the gastrointestinal tract, is critically involved in cancer [2, 3, 4]. Microbiota dysbiosis, a disruption in these microbial communities, has been reported to be associated with the development and progression of various types of cancer [5, 6]. In contrast, the increase in population of specific beneficial bacterial species within the gut ecosystem has been shown to strengthen the host's immune system and to improve immune surveillance in the tumor microenvironment [7, 8, 9, 10]. This was observed not only in gastrointestinal tumors, but also in cancer types with a high degree of immune infiltration, such as lung cancer and melanoma [11, 12, 13]. Notably, certain gut microbiota can also affect the effectiveness of anticancer treatments, particularly immunotherapy, by influencing the host's immune response and drug metabolism. These highlight the necessity of taking microbiota into consideration as a crucial player in the disease landscape while developing novel therapeutic approaches for cancer treatment.

Akkermansia (Akk) has emerged as a key player in gut microbiota research, with growing evidence highlighting its influence on cancer immunology and immunotherapy outcomes [4, 9, 14, 15]. Akk is a mucin‐degrading bacterium that helps maintain intestinal barrier integrity and supports gut homeostasis [16]. In oncology, Akk has been reported to exert multiple beneficial effects. Akk was discovered colonizing in lung tumor tissues and suppressing tumorigenesis by modulating the tumoral microbiota and reprogramming cancer cell metabolism [17]. Furthermore, the Akk outer membrane protein Amuc_1100 was shown to enhance tumor‐infiltrating CD8+ T cell recruitment for countering cancer cell immune evasion [18, 19]. Importantly, various studies have revealed that the high abundance of Akk in intestinal microbiota is associated with good responses to cancer immune checkpoint inhibitors [20, 21]. Derosa et al. discovered that the abundance of Akk in gut microbiota can be used as a potential predictor of clinical response to PD‐1 blockade in patients with non‐small‐cell lung cancer [9]. In melanoma patients responding to PD‐1 blockade, higher Akk abundance correlated with improved outcomes, and fecal microbiota transplantation from responders enhanced anti‐tumor immunity and anti‐PD‐L1 efficacy in germ‐free mice [12]. Taken together, Akk orchestrates multifaceted immunomodulatory effects that potentiate cancer therapy efficacy, positioning targeted modulation of gut Akk abundance as a promising therapeutic avenue in oncology. However, the clinical translation of Akk‐based therapies still faces fundamental challenges, including production and delivery, sustainable intestine colonization, inter‐patient variability, safety risks in immunocompromised populations, and regulatory complexities for live organisms [22]. Therefore, there is a critical demand for innovative approaches that can effectively and sustainably augment the population of Akk within the gastrointestinal tract.

Traditional herbal medicine serves as a valuable source of novel therapeutic agents in oncology. With oral administration being the primary method for these medicines, their therapeutic functions have been found to significantly involve the participation of gut microbiota [23, 24, 25]. Icariin, the primary active component of Epimedium, exerts notable inhibitory effects against a wide spectrum of cancer types [26, 27, 28]. Other than its direct cytotoxicity on tumor cells, accumulating evidence indicates that icariin's immunomodulatory role in the tumor microenvironment significantly contributes to its antitumor activities in vivo [26]. Icariin and its metabolite icaritin have been prominently recognized for activating CD8+ T cells and facilitating their infiltration into TME [29, 30, 31]. Additionally, icariin was also found to reduce populations of immunosuppressive cells like M2 macrophages, and inhibit the production of immune suppressive factors such as TNF‐alpha [28, 32], thereby reversing immune suppression and promoting anti‐tumor immunity. Moreover, the involvement of gut microbiota interaction in icariin's pharmacological functions was also reported [33, 34]. The combination of icariin and curcumol impedes the progression of prostate cancer by modulating the gut microbiota and activating CD8+ T cells through the DNMT1/IGFBP2 axis [31]. Icariin also conferred neuroprotection via gut microbiome modulation, effectively reversing microbial dysbiosis in both the transgenic AD mouse model and naturally aged mice [35]. While icariin demonstrates both immunomodulatory and microbiota‐altering properties, their mechanistic interplay led by icariin in cancer remains poorly understood.

In this study, we demonstrated that the antitumor activity of icariin is closely linked to immune modulation regulated by icariin‐enriched Akk. Oral administration of icariin significantly enriched the abundance of gut Akk in two tumor‐bearing and non‐tumor mice, regardless of whether the mice were immunocompetent or immunodeficient. Moreover, in vitro assays confirmed that icariin promoted Akk enrichment in both murine and human fecal microbiota cultures. We found that icariin promoted Akk growth in a mucin‐dependent manner. Icariin regulated mucin metabolism‐related transcriptomic and metabolomic pathways to promote Akk growth. Transcriptomic and metabolomic analyses revealed that icariin enhanced mucin‐associated metabolic pathways to support Akk growth. Mechanistically, icariin enhanced the activity of the N‐acetylgalactosaminidase Amuc_0920 by stabilizing key residues at the binding sites for the substrate GalNAc. Functionally, metagenomic analysis and single‐cell RNA sequencing further found that icariin‐induced Akk enrichment significantly enhanced the immune profile within the tumor microenvironment, characterized by the reduction of the TNFα pathway and the increase of the CCL3 signaling pathway in CD8+ T cells. Furthermore, icariin‐induced Akk enrichment in the gut microbiota effectively enhanced the efficacy of anti‐PD‐1 therapy in LLC and B16 mouse models. This work demonstrates the potential of icariin as a potent prebiotic that specifically enriches Akk in the gut microbiota, thereby exerting significant antitumor effects and enhancing the efficacy of PD‐1 blockade therapy (Figure 11).

## 2 Results

### 2.1 Gut Microbiota is a Critical Mediator of Icariin's Tumor‐Suppressive Activities in LLC and B16 Mouse Models

**FIGURE 1:**
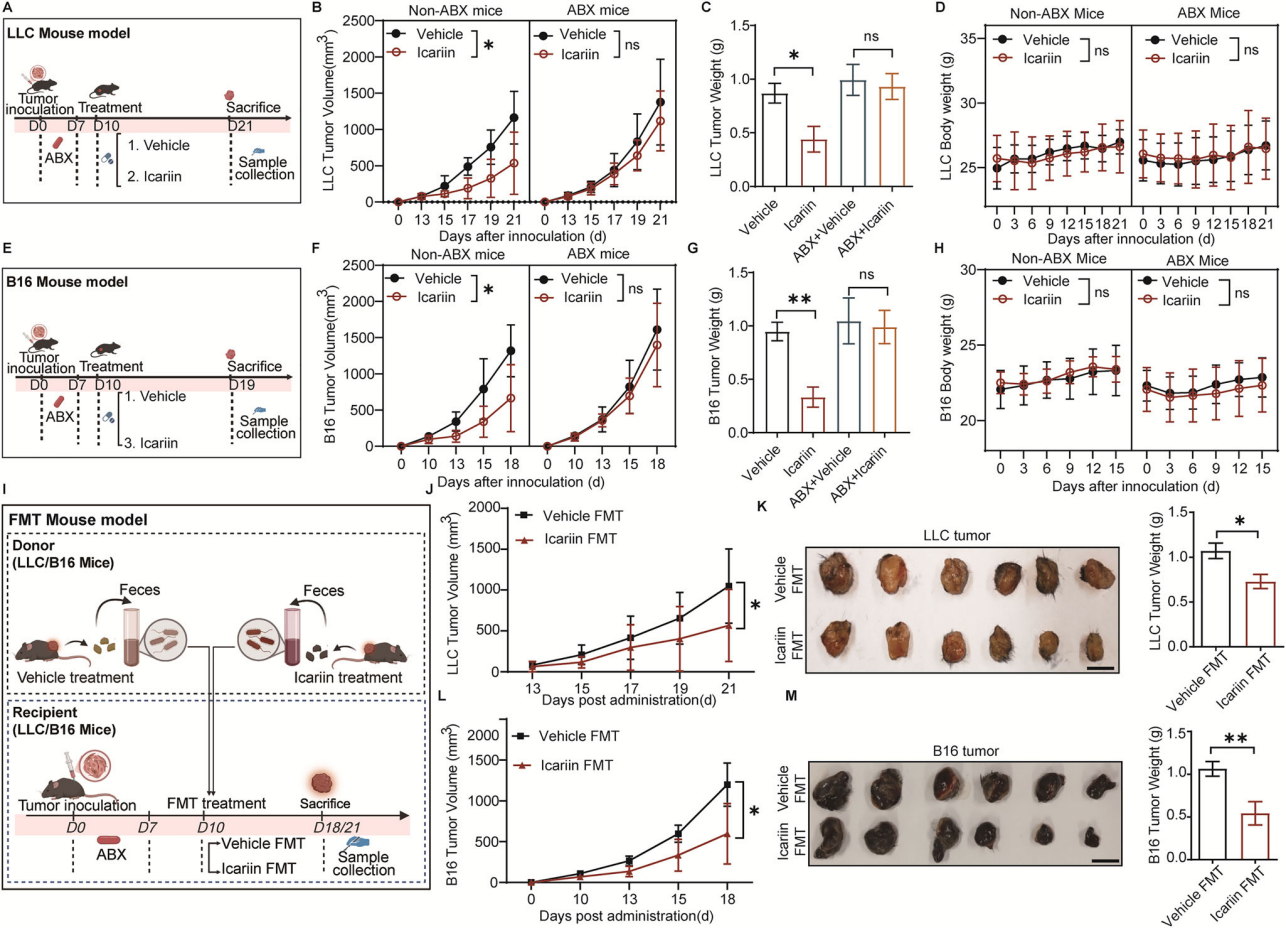
Gut microbiota mediated icariin's antitumor efficacy in LLC and B16 mouse models. (A) Experimental design of icariin treatment in LLC tumor‐bearing mice with (ABX) or without (non‐ABX) antibiotic pretreatment (*n* = 6/group). (B) Tumor growth curve in non‐ABX and ABX LLC tumor‐bearing mice receiving with icariin (70 mg/kg) or vehicle. (C) Tumor weight in non‐ABX and ABX LLC tumor‐bearing mice receiving with icariin or vehicle. (D) Body weight for biological safety in non‐ABX and ABX LLC tumor‐bearing mice receiving with icariin or vehicle. (E) Experimental design of icariin treatment in non‐ABX and ABX B16 tumor‐bearing mice (*n* = 6/group). (F) Tumor growth curve in non‐ABX and ABX B16 tumor‐bearing mice receiving with icariin or vehicle (70 mg/kg). (G) Tumor weight in non‐ABX and ABX B16 tumor‐bearing mice treated with icariin or vehicle. (H) Body weight for biological safety in non‐ABX and ABX B16 tumor‐bearing mice receiving with icariin or vehicle. (I) Experimental design for fecal microbiota transplantation (FMT) in LLC and B16 mouse models (*n* = 6/group). (J) Tumor growth curve in LLC tumor‐bearing mice receiving with icariin‐FMT or vehicle‐FMT. (K) Tumor photographs and statistics of LLC tumor‐bearing mice receiving with icariin‐FMT or vehicle‐FMT. (L) Tumor growth curve in B16 tumor‐bearing mice receiving with icariin‐FMT or vehicle‐FMT, scale bar: 1 cm. (M) Tumor photographs and statistics of B16 tumor‐bearing mice receiving with icariin‐FMT or vehicle‐FMT, scale bar: 1 cm. Two‐tailed unpaired t‐test: ^*^ *p* < 0.05; ^**^ *p* < 0.01.

Lung cancer and melanoma have been extensively studied for their complex interaction with the gut microbiota [36, 37]. Here, we employed murine allograft tumor models of Lewis lung carcinoma (LLC) and B16‐F10 melanoma (B16), with or without the antibiotic‐induced microbiota‐depletion pretreatment (ABX) (Figure 1A,E). In the LLC tumor model, icariin treatment significantly reduced tumor burden in non‐ABX LLC mice, but failed to inhibit tumor progression in ABX LLC mice (Figure 1B,C; Figure S2A,C). Similarly, in the B16 tumor model, icariin markedly suppressed tumor growth; this effect was substantially weakened in ABX B16 mice (Figure 1F,G; Figure S2B,D). No significant change in body weight was observed in these mice after icariin treatment (Figure 1D,H). The decreased antitumor activity of icariin after microbiota depletion in both tumor models strongly suggested that gut microbiota might be a critical mediator for icariin's biological function. Therefore, a fecal microbiota transplantation (FMT) experiment was subsequently conducted in LLC and B16 mouse models. Fecal samples were collected from tumor‐bearing donor mice treated with either vehicle or icariin, and these samples were transplanted into ABX tumor‐bearing recipient mice (Figure 1I). In the LLC tumor‐bearing mice, the tumor growth curve reflected that icariin‐FMT recipients significantly attenuated tumor progression compared to vehicle‐FMT recipients (Figure 1J). Final tumor burden measurement also revealed a substantial reduction in tumor weight among the icariin‐FMT recipients (Figure 1K). In the B16 tumor‐bearing mice, tumor volume monitoring also indicated a pronounced regression in icariin‐FMT recipients compared to vehicle‐FMT recipients (Figure 1L). Final tumor weight further showed icariin‐FMT treatment had stronger anti‐tumor effects than vehicle‐FMT (Figure 1M). These results demonstrated the significant impact of gut microbiota on the tumor suppression activities of icariin.

### 2.2 Icariin Reshaped Gut Microbiota Composition With Enrichment of Akk in LLC and B16 Mouse Models

**FIGURE 2:**
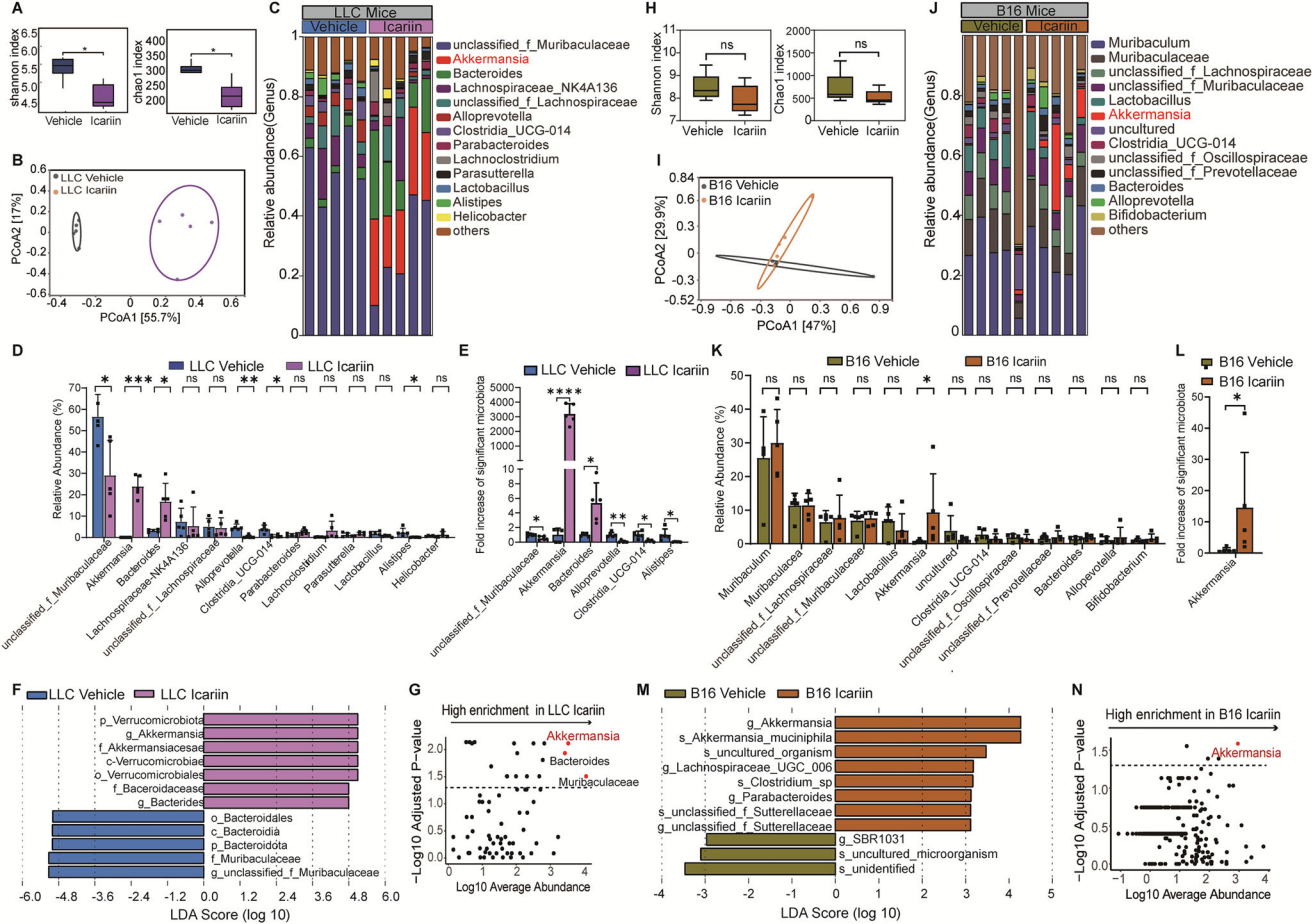
Icariin reshaped gut microbiota composition and preferentially enriched the probiotic bacterium Akk in LLC and B16 mouse models. (A) α diversity index analysis for the richness and diversity of gut microbiota in LLC tumor‐bearing mice receiving with icariin or vehicle (*n* = 5/group). (B) Principal component analysis (PCA) for β diversity of gut microbiota in LLC tumor‐bearing mice. (C) Genus‐level microbial composition in LLC tumor‐bearing mice. (D) Bar plot for the most abundant bacterial genera in LLC tumor‐bearing mice. (E) Fold change of significant genera in LLC tumor‐bearing mice. (F) Linear discriminant analysis (LDA) with effect size measurement for significantly abundant taxa in LLC tumor‐bearing mice, with icariin‐enriched taxa (purple, positive LDA scores) and vehicle‐enriched taxa (blue, negative scores). (G) Volcano plot of differentially abundant genus‐level OTUs in LLC tumor‐bearing mice, with a horizontal dotted line indicating the *p* = 0.05 threshold. (H) α diversity index analysis for the richness and diversity of gut microbiota in B16 tumor‐bearing mice receiving icariin or vehicle (*n* = 5/group). (I) Principal component analysis (PCA) for β diversity of gut microbiota in B16 tumor‐bearing mice. (J) Genus‐level microbial composition in B16 tumor‐bearing mice. (K) Bar plot for the most abundant bacterial genera in B16 tumor‐bearing mice. (L) Fold change of significant genera in B16 tumor‐bearing mice. (M) Linear discriminant analysis (LDA) with effect size measurement for significantly abundant taxa in B16 tumor‐bearing mice, with icariin‐enriched taxa (orange, positive LDA scores) and vehicle‐enriched taxa (green, negative LDA scores). (N) Volcano plot of differentially abundant genus‐level OTUs in B16 tumor‐bearing mice, with a horizontal dotted line indicating the p = 0.05 threshold. Two‐tailed unpaired t‐test: ^*^ *p* < 0.05; ^**^ *p* < 0.01. ^***^ *p* < 0.001.

To investigate how icariin influences gut microbiota composition in tumor‐bearing mice, we employed 16S rRNA sequencing on fecal samples collected from LLC and B16 mouse models, both treated and untreated with icariin. In the LLC mouse model, alpha diversity analysis based on the Shannon and Chao1 index demonstrated that the icariin group significantly reduced the community richness and evenness of the gut microbiota, suggesting that certain dominant bacterial populations might have expanded, thereby leading to a decrease in microbial diversity (Figure 2A). Furthermore, principal coordinate analysis (PCoA) of beta‐diversity demonstrated that the microbial communities in the icariin‐treated group clustered separately from those in the vehicle group, indicating that icariin treatment significantly reshaped the gut microbiota composition in the LLC mouse model (Figure 2B). Barplots of microbial communities subsequently displayed variations in the composition and abundance of the most dominant microbes among the samples. At the genus level, Akkermansia (Akk) showed a remarkable increase in the LLC icariin group, with its relative abundance rising by nearly 3000‐fold compared to that in the vehicle group (Figure 2C–E). Besides, a significant increase in *Bacteroides*, and markedly decreases in *Muribaculaceae, Alloprevotella, Clostridia and Alistipes* were also detected (Figure 2C–E). Linear discriminant analysis (LDA), which quantifies the relative abundance and statistical significance of different bacterial between groups, further revealed significant changes in gut microbiota composition between the LLC vehicle and LLC icariin groups. Notably, nearly the full taxonomic hierarchy of Akk (*p_Verrucomicrobiota*, *c_Verrucomicrobiotiae, o_Verrucomicrobiales, f_Akkermansiacesae, g_Akkermansia*) all exhibited significantly higher LDA scores than the LLC vehicle group (Figure 2F). Moreover, volcano plot analysis also showed significant enrichment of several genera in the LLC icariin group, with Akk showing the most substantial increase in abundance (Figure 2G).

In the B16 mouse model, alpha diversity analysis revealed a trend toward decreased richness (Chao1 index) and diversity (Shannon index) in the gut microbiota of the icariin group (Figure 2H). PCoA analysis demonstrated a clear separation in microbial community composition between the icariin and vehicle groups (Figure 2I). Genus‐level microbial community analysis revealed that icariin treatment induced a 15‐fold increase in the relative abundance of Akk, which was the only genus among the top fifteen most abundant taxa showing significant change (Figure 2J–L). The highest LDA scores in the icariin group were also observed for Akk at the genus and species level (*g_Akkermansia and s_Akkermansia muciniphila*) (Figure 2M). Volcano plot analysis further confirmed Akk as the most differentially abundant genus in the B16 icariin group compared to the B16 vehicle group (Figure 2N).

### 2.3 Icariin Potently Elevated Akk Abundance in Non‐Tumor‐Bearing Mice Regardless of Host Immune Status

**FIGURE 3:**
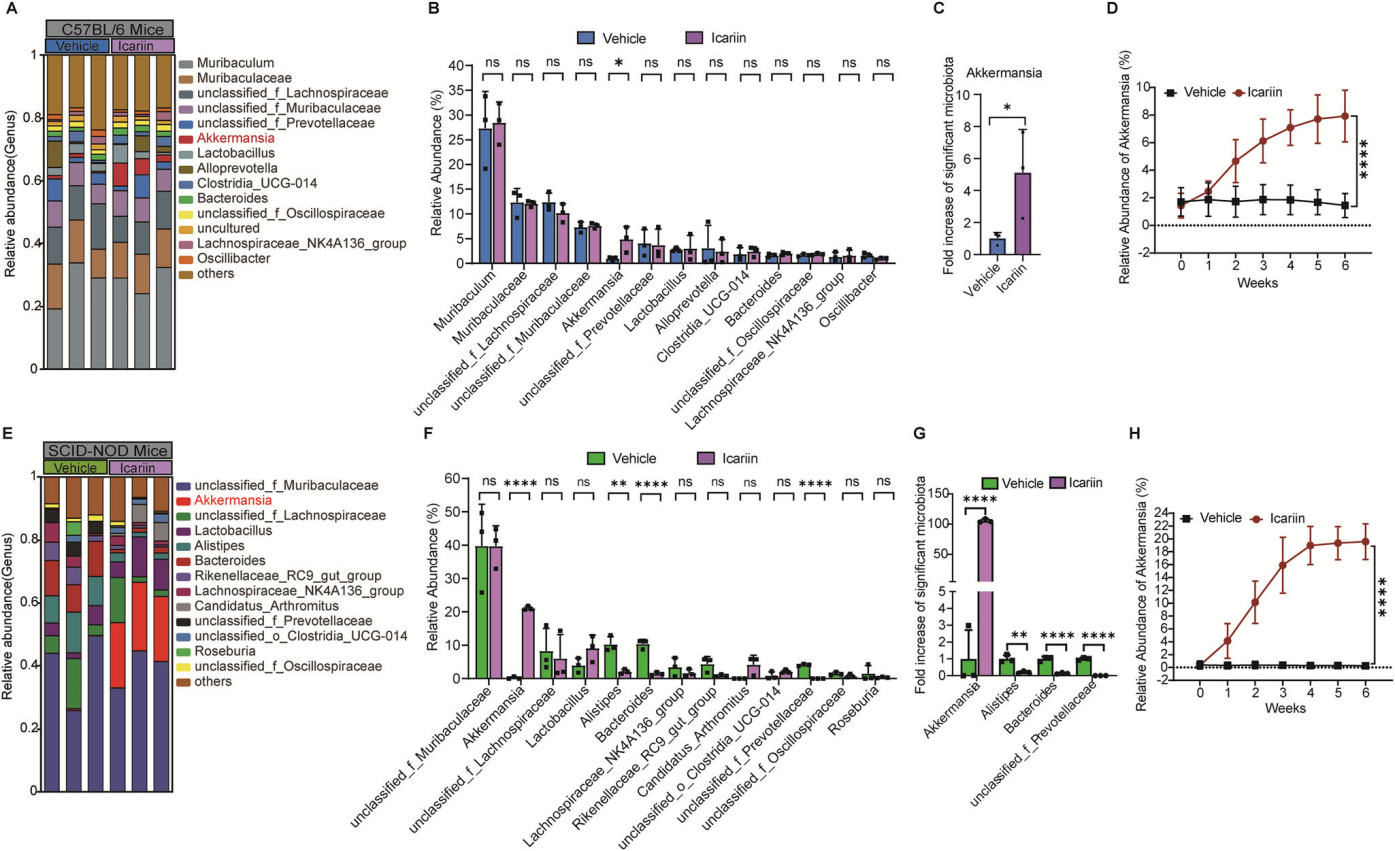
Icariin specifically enriched Akk abundance in both immune‐competent and immune‐compromised mice. (A) Genus‐level microbial composition in healthy C57BL/6 mice receiving with vehicle or icariin (*n* = 3/group). (B) Bar plot for the most abundant bacterial genera in healthy C57BL/6 mice. (C) Fold change of significant genera in healthy C57BL/6 mice. (D) Relative abundance of fecal Akk in healthy C57BL/6 mice receiving with vehicle or icariin for 6 weeks by qPCR analysis (*n* = 6/group). (E) Genus‐level microbial composition in immunodeficient SCID‐NOD mice receiving with vehicle or icariin (*n* = 3/group). (F) Bar plot for the most abundant bacterial genera in immunodeficient SCID‐NOD mice. (G) Fold change of significant genera in immunodeficient SCID‐NOD mice. (H) Relative abundance of fecal Akk in immunodeficient SCID‐NOD mice receiving with vehicle or icariin for 6 weeks by qPCR analysis(*n* = 6/group). Two‐tailed unpaired t‐test: ^*^ *p* < 0.05; ^**^ *p* < 0.01; ^***^ *p*<0.001.

As icariin induced dramatic increases of Akk abundance in two tumor‐bearing mouse models, we wonder whether this effect of icariin on gut microbiota is related to the tumor. Therefore, we performed 16S rRNA sequencing on fecal samples from C57BL/6 mice that were not inoculated with tumors and treated with either vehicle or icariin. Microbial community analysis at the genus level revealed that icariin treatment significantly increased the relative abundance of Akk by 5‐fold, making it the only genus among the most abundant taxa to exhibit such a pronounced increase (Figure 3A–C). Furthermore, qPCR analysis confirmed that icariin treatment enhanced the relative abundance of Akk in the fecal microbiota of C57BL/6 mice over a period of 6 weeks (Figure 3D).

Subsequently, we employed immunodeficient SCID‐NOD mice to investigate whether icariin‐induced Akk enrichment in the gut microbiota is affected by the host immune system. In SCID‐NOD mice, although baseline Akk abundance exhibited a little inter‐individual variation, icariin treatment also significantly elevated its relative abundance to about 20%, representing an increase of over 100‐fold (Figure 3E–G). Notably, besides a significant enrichment of Akk, other dominant bacterial genera such as Alistipes, Bacteroides, and Prevotellaceas showed a declining trend in SCID‐NOD mice (Figure 3F,G). qPCR analysis further confirmed a sustained increase in fecal Akk levels in SCID‐NOD mice, suggesting that icariin‐mediated Akk enrichment may be independent of adaptive immunity (Figure 3H).

### 2.4 Icariin Promoted Akk Growth in a Mucin‐Dependent Manner In Vitro

**FIGURE 4:**
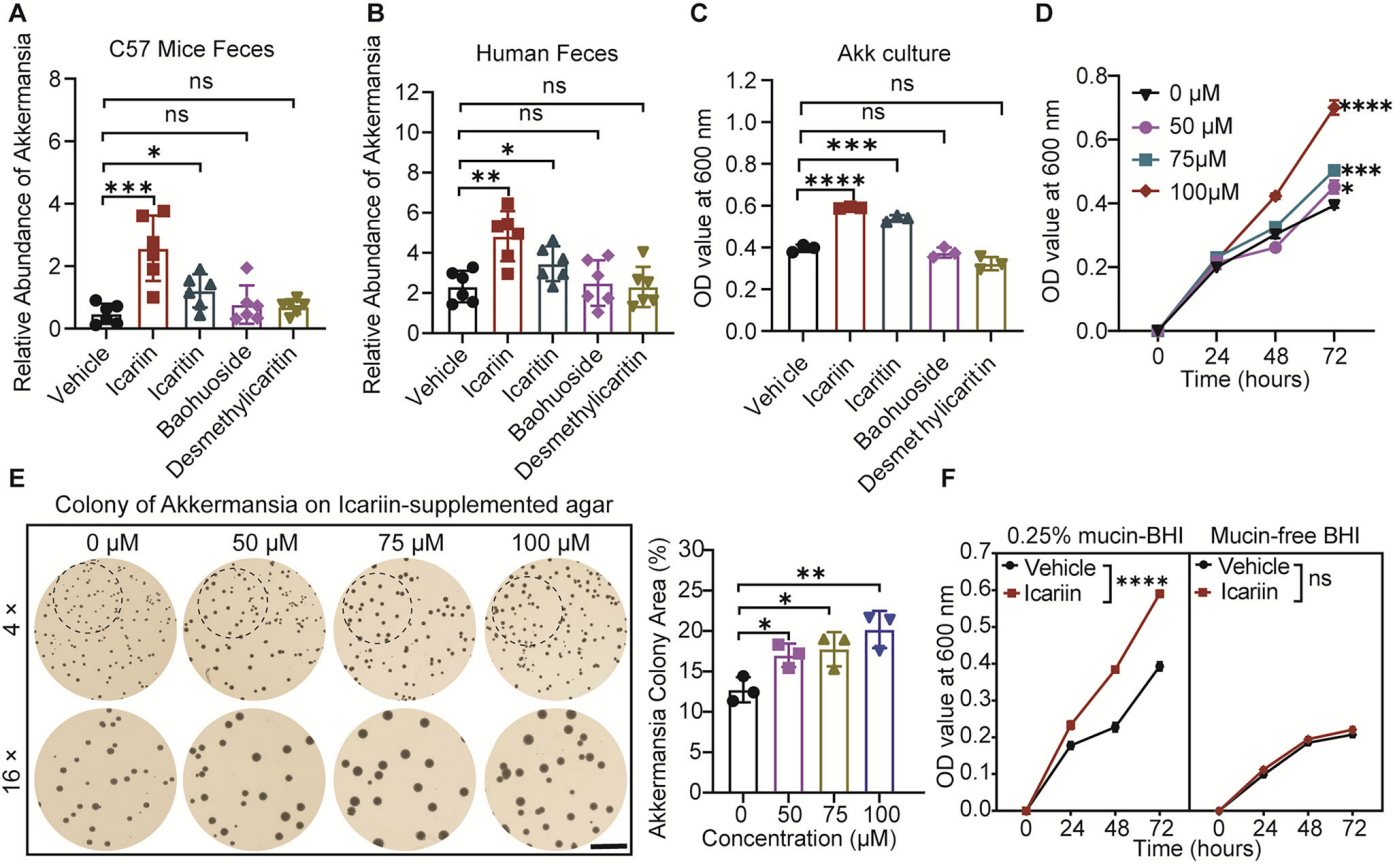
Icariin promoted Akk growth in a mucin‐dependent manner in vitro. (A) qPCR analysis of Akk relative abundance in C57BL/6 mouse fecal microbiota cultured with 100 µm icariin or its metabolites (icaritin, baohuoside, desmethylicaritin). (B) qPCR analysis of Akk relative abundance in human fecal microbiota cultured with 100 µm icariin or its metabolites (icaritin, baohuoside, desmethylicaritin). (C) Growth barplot analysis (OD600) of Akk cultured in 0.25% mucin‐BHI medium with 100 µm icariin or its metabolites (icaritin, baohuoside, desmethylicaritin). (D) Growth curve (OD600) of Akk cultured in 0.25% mucin‐BHI medium supplemented with icariin (0–100 µm). (E) Representative colonies and statistical analysis of Akk cultured on icariin‐supplemented 0.25% mucin‐BHI agar plates (0–100 µm), with 16× magnified images corresponding to the dashed circles in the 4× images, scale bar: 1 cm. (F) Growth curves (OD600) of Akk cultured in 0.25% mucin‐BHI and mucin‐free BHI media with either vehicle or 100 µm icariin. Two‐tailed unpaired t‐test: ^*^ *p* < 0.05; ^**^ *p* < 0.01; ^***^ *p* < 0.001.

Icariin is metabolized in the gut to produce bioactive metabolites such as icaritin, baohuoside, and desmethylicaritin (Figure S1). To investigate if icariin or its metabolites modulates Akk, we collected fecal microbiota from C57BL/6 mice and human volunteers, and cultured them with icariin and its metabolites in vitro. Notably, qPCR analysis revealed that icariin and icaritin (the metabolite) group significantly increased the relative abundance of Akk in the fecal gut microbiota of both mice and humans, with icariin showing a more pronounced effect (Figure 4A,B). To further explore the growth‐promoting effects of icariin on Akk, we treated the pure Akk strain (ATCC BAA‐835) with icariin and its metabolite in 0.25% mucin‐supplemented BHI medium, and observed that both icariin and icaritin significantly promoted Akk growth in vitro (Figure 4C). We then treated the Akk strain with varying concentrations of icariin (0–100 µm). Growth curve analysis based on OD600 nm revealed that icariin significantly enhanced Akk growth in both dose‐ and time‐dependent manners (Figure 4D). When Akk was cultured on 0.25% mucin‐supplemented BHI agar plates with varying icariin concentrations (0–100 µm), the number of colony‐forming units (CFUs) remained unchanged (Figure S3). Since colony area, an index for biomass accumulation, more appropriately reflects the effect of icariin, we used it to assess bacterial growth. The icariin group markedly increased the colony area in a concentration‐dependent manner (Figure 4E). Given that Akk specializes in degrading mucin (e.g., MUC2) as its primary carbon and energy source to sustain its growth [38]. To assess whether mucin is required for the growth‐promoting effect of icariin on Akk, we cultured the Akk in BHI medium with or without 0.25% mucin supplementation in the presence of icariin. Growth curve analysis showed that the promoting effect of icariin on Akk growth was completely abolished under mucin‐free conditions (Figure 4F). These in vitro findings indicated that icariin promoted Akk. growth in a mucin‐dependent manner.

### 2.5 Transcriptomic and Metabolomic Analyses Revealed That Icariin Enhanced Mucin‐Associated Metabolic Pathways to Support Akk Growth

**FIGURE 5:**
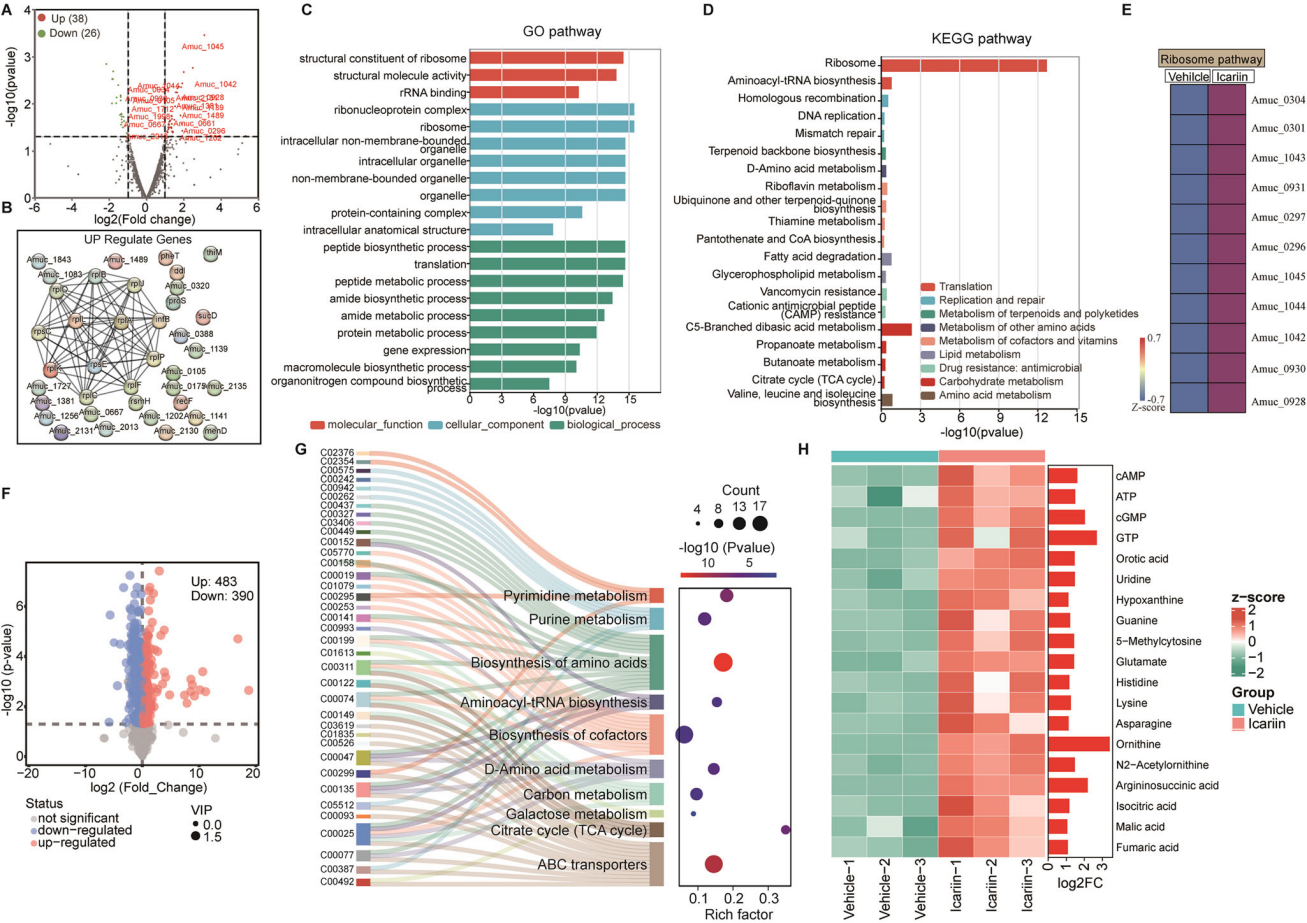
Transcriptomic and metabolomic analyses revealed that icariin enhanced mucin‐associated metabolic pathways to support Akk growth. (A) Volcano plot of differentially expressed genes in Akk cultured with 0.25% mucin‐BHI medium after 72 h treatment with vehicle or 100 µm icariin by RNA seq, red dots represent upregulated genes. (B) Gene co‐expression network of significant genes of Akk by RNA seq. (C) Gene Ontology (GO) analysis of significant genes of Akk by RNA seq. (D) KEGG pathway analysis of significant genes of Akk by RNA seq. (E) Z‐score of ribosomal pathway genes of Akk by RNA seq. (F) Volcano plot of differentially metabolites in Akk cultured with 0.25% mucin‐BHI medium after 72 h treatment with vehicle or 100 µm icariin by transcriptomic analysis. Red dots represent upregulated metabolites, green dots represent downregulated metabolites. (G) Sankey‐bubble plot of KEGG metabolic pathways enrichment of Akk. Connecting arcs indicate the associations between key metabolic pathways and related metabolites. (H) Heatmap‐bar plot of key metabolites in Akk cultured with 0.25% mucin‐BHI medium after 72 h treatment with vehicle or 100 µm icariin by transcriptomic analysis. Two‐tailed unpaired t‐test: ^*^ *p* < 0.05; ^**^ *p* < 0.01; ^***^ *p* < 0.001.

To elucidate the molecular mechanisms underlying icariin‐mediated modulation of Akk growth in mucin‐dependent conditions, we performed RNA sequencing to analyze transcriptomic changes in Akk cultured in 0.25% mucin‐BHI medium, comparing vehicle‐treated with 100 µm icariin‐treated groups. Transcriptomic analysis identified 64 significantly differentially expressed genes (DEGs) (|log2FC| >1, padj <0.05) in the icariin group, with 38 genes upregulated and 26 downregulated (Figure 5A,B). Gene Ontology (GO) enrichment analysis revealed that the majority of DEGs were enriched in biological process (BP) terms associated with gene expression, translation, and protein biosynthesis and metabolism, including peptide/amide metabolic processes, indicating icariin might regulate protein synthesis and metabolic capacity to support Akk growth (Figure 5C).

KEGG pathway analysis was further performed to assess the functional impact of icariin on Akk DEGs. The analysis showed significant enrichment in translation (ribosome biogenesis, aminoacyl‐tRNA biosynthesis) and DNA maintenance (homologous recombination, replication, mismatch repair) pathways (Figure 5D). Notably, the icariin group significantly upregulated ribosomal genes (Amuc_0928–0931, Amuc_1042–1145, Amuc_0296–0297, Amuc_0301, Amuc_0304) and genes linked to aminoacyl‐tRNA biosynthesis (Amuc_0034, Amuc_1998), suggesting a potential enhancement of protein synthesis capacity (Figure 5B,E). Beyond enhancing transcriptional activity, the icariin group significantly upregulated genes associated with key metabolic pathways, including Amino acid metabolism (Amuc_0667, Amuc_2013, Amuc_0661), Carbohydrate metabolism (Amuc_1712, Amuc_0667), and Glycan biosynthesis and metabolism (Amuc_0661) (Figure 5B,E). The upregulation of these metabolic pathway genes enhanced Akk's capacity to metabolize degradation products from both the polypeptide backbone and glycan chains of mucin, thereby enabling more efficient utilization of amino acids and monosaccharides released from mucin for energy production and biosynthesis. These transcriptomic results indicated that icariin promoted Akk's mucin catabolic efficiency and metabolic adaptation through coordinated gene expression changes.

Next, we further employed metabolomic analysis to investigate how icariin regulated key metabolic pathways in Akk. We cultured Akk in 0.25% mucin‐BHI medium supplemented with either 100 µm icariin or vehicle, and observed marked metabolic alterations induced by icariin. Compared to the vehicle group, a total of 873 metabolites were found to be significantly changed (|log2FC| > 1, padj < 0.05), including 483 upregulated and 390 downregulated metabolites (Figure 5F). Functional enrichment analysis of these differentially expressed metabolites showed that purine and pyrimidine metabolism, which are crucial pathways for nucleic acid synthesis, were the most significantly affected by icariin (Figure 5G). Further analysis revealed that key metabolic intermediates such as lactosyl acid, guanosine, hypoxanthine, guanine, and 5‐methylcytosine, which serve as key precursors for nucleic acid synthesis, were significantly elevated in the icariin group (Figure 5H). This indicated that Akk could more efficiently utilize the substrates released from mucin degradation for nucleic acid biosynthesis. Additionally, the icariin group markedly increased ATP and GTP levels within the purine and pyrimidine metabolic pathways, suggesting that icariin not only promoted the energy conversion of mucin‐derived metabolites but also enhanced energy storage capacity in Akk (Figure 5H). Importantly, icariin also enhanced amino acid biosynthesis and aminoacyl‐tRNA biosynthesis pathways (Figure 5G), which promoted Akk's capacity for protein synthesis (Figure 5G). Heatmap‐barplot analysis further showed significant increases in essential amino acids required for protein synthesis, such as glutamate, histidine, and lysine, suggesting that icariin facilitated the utilization of mucin catabolism for protein biosynthesis in Akk (Figure 5H).

Moreover, the icariin group led to a marked increase in cyclic AMP (cAMP) and cyclic GMP (cGMP) levels, which serve as important secondary messengers. In bacteria, cAMP and cGMP can respond to the abundance of mucin‐derived substrates and activate key metabolic and adaptive pathways, including the tricarboxylic acid (TCA) cycle and amino acid metabolism [39, 40]. Pathway enrichment analysis demonstrated that icariin significantly promoted amino acid metabolism and the TCA cycle (Figure 5G), with corresponding increases in TCA cycle intermediates such as isocitric acid, malic acid, and fumaric acid (Figure 5H), further promoting energy metabolism. Taken together, icariin promoted transcriptomic and metabolomic pathways related to mucin metabolism, accelerating the catabolism and efficient utilization of mucin‐derived substrates. This metabolic reprogramming enhanced the synthesis of nucleic acids and proteins, as well as central energy metabolism to support Akk growth.

### 2.6 Icariin Enhanced the Activity of the N‐acetylgalactosaminidase Amuc_0920 by Stabilizing Key Residues at the Binding Sites for the Substrate GalNAc

**FIGURE 6:**
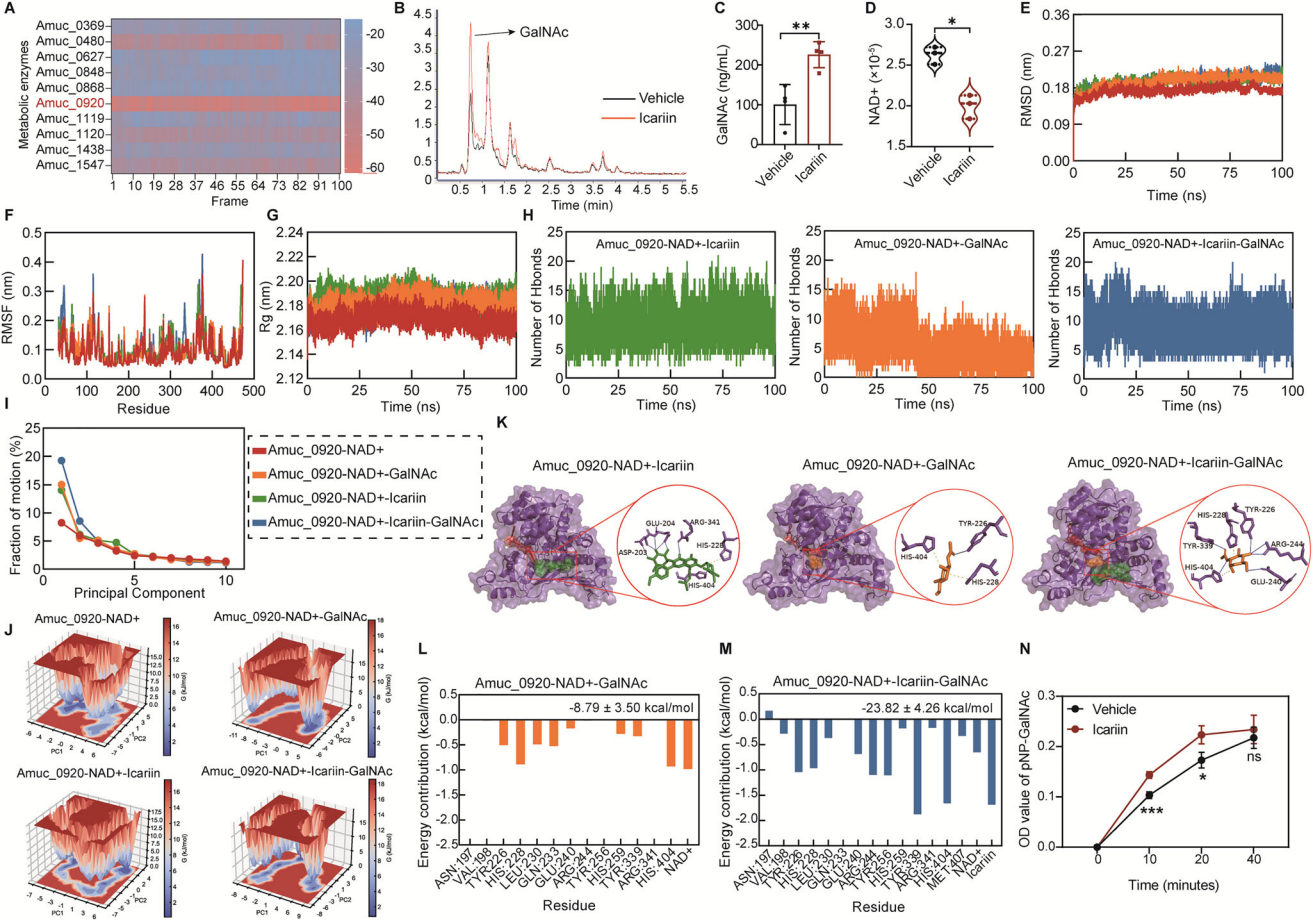
Icariin enhanced the activity of the N‐acetylgalactosaminidase Amuc_0920 by stabilizing key residues at the binding sites for the substrate GalNAc. (A) The heatmap of the changes in binding free energy between the ten enzyme proteins and Icariin over the interval of 100 simulation frames. (B, C) HPLC‐MS profiling (B) and quantification (C) of mucin‐derived N‐acetylgalactosamine (GalNAc) in Akk cultured in 0.25% mucin‐BHI medium with either vehicle or 100 µm icariin. (D) Metabolomic analysis of NAD+ levels in Akk cultured in 0.25% mucin‐BHI medium with either vehicle or 100 µm icariin. (E) Root mean square deviation (RMSD) analysis of Amuc_0920‐NAD+ complexes with different ligands during 100 ns molecular dynamics simulations (MDs). The RMSD values (nm) are shown for Amuc_0920‐NAD+ (red), Amuc_0920‐NAD+‐GalNAc (orange), Amuc_0920‐NAD+‐Icariin (green), and Amuc_0920‐NAD+‐Icariin‐GalNAc (blue). (F) Root mean square fluctuation (RMSF) analysis of each residue in Amuc_0920‐NAD+ complexes with different ligands from MDs. RMSF values (nm) are shown for Amuc_0920‐NAD+ (red), Amuc_0920‐NAD+‐GalNAc (orange), Amuc_0920‐NAD+‐Icariin (green), and Amuc_0920‐NAD+‐Icariin‐GalNAc (blue). (G) Radius of gyration (Rg) analysis of Amuc_0920‐NAD+ complexes with different ligands during 100 ns MDs. Rg values (nm) are shown for Amuc_0920‐NAD+ (red), Amuc_0920‐NAD+‐GalNAc (orange), Amuc_0920‐NAD+‐Icariin (green), and Amuc_0920‐NAD+‐Icariin‐GalNAc (blue). (H) Time evolution of the number of hydrogen bonds formed between ligands and protein in different Amuc_0920‐NAD+ complex systems during 100 ns MDs. The number of hydrogen bonds (Hbnum) is shown for hydrogen bonds between Icariin and Amuc_0920‐NAD+ in the Amuc_0920‐NAD+‐Icariin system (green), hydrogen bonds between GalNAc and Amuc_0920‐NAD+ in the Amuc_0920‐NAD+‐GalNAc system (orange), and hydrogen bonds between GalNAc and Amuc_0920‐NAD+‐Icariin in the Amuc_0920‐NAD+‐Icariin‐GalNAc system (blue). (I) Principal component analysis (PCA) of the MDs trajectories for Amuc_0920‐NAD+ complexes with different ligands. The plot shows the fraction of motion (%) explained by the first 10 principal components for Amuc_0920‐NAD+ (red), Amuc_0920‐NAD+‐GalNAc (orange), Amuc_0920‐NAD+‐Icariin (green), and Amuc_0920‐NAD+‐Icariin‐GalNAc (blue). (J) Free energy landscapes (FEL) of different Amuc_0920‐NAD+ complex systems projected onto the first two principal components (PC1 and PC2) from PCA analysis of MDs trajectories. Shown are: Amuc_0920‐NAD+ (top left), Amuc_0920‐NAD+‐GalNAc (top right), Amuc_0920‐NAD+‐Icariin (bottom left), and Amuc_0920‐NAD+‐Icariin‐GalNAc (bottom right). Free energy values (kcal/mol) are indicated by the color scale. (K) Binding modes of Icariin (green) and/or GalNAc (orange) in different Amuc_0920‐NAD+ complex systems, with key interacting residues highlighted. Left: Amuc_0920‐NAD+‐Icariin; center: Amuc_0920‐NAD+‐GalNAc; right: Amuc_0920‐NAD+‐Icariin‐GalNAc. The overall protein structure is shown as a purple surface, and close‐up views of the ligand‐binding sites with interacting residues are provided in the circles. (L) Per‐residue energy contributions (kcal/mol) to GalNAc binding in the Amuc_0920‐NAD+‐GalNAc complex based on MDs. The total binding energy is indicated above the bar plot. (M) Per‐residue energy contributions (kcal/mol) to GalNAc binding in the Amuc_0920‐NAD+‐Icarrin‐GalNAc complex based on MDs. The total binding energy is indicated above the bar plot. N. N‐Acetyl‐galactosaminidase activity toward pNP‐GalNAc in Akk cultured with vehicle or 100 µm icariin. Two‐tailed unpaired t‐test: ^*^ *p* < 0.05; ^**^ *p* < 0.01; ^***^ *p* < 0.001.

Akk utilizes an enzymatic system comprising sulfatases, sialidases, fucosidases, and glycoside hydrolases to degrade mucin, thereby providing essential nutrients required for its growth [41]. To investigate whether icariin regulates mucin‐degrading enzymes derived from Akk, we performed AlphaFold3 to predict the complex structures of icariin and ten mucin‐degrading enzymes with known crystal structures, and calculated the binding free energies between icariin and these enzymes. Icariin showed a significantly stronger predicted binding affinity to Amuc_0920 than to the other mucin‐degrading enzymes (Figure 6A; Figure S4A). As a member of glycoside hydrolase family 109 (GH109), Amuc‐0920 specifically catalyzes the hydrolysis of N‐acetylgalactosamine (GalNAc) glycosidic bonds, which is crucial in the degradation of complex polysaccharides and carbohydrate metabolism [41]. To investigate the regulatory effects of icariin on the enzymatic activity of Amuc_0920, we employed liquid chromatography‐triple quadrupole mass spectrometry (LC‐QqQ‐MS) to quantify the production of GalNAc. LC‐QqQ‐MS results showed that the GalNAc levels derived from mucin degradation in the icariin‐treated Akk cultures were significantly higher than those in the vehicle group (Figure 6B,C). Furthermore, GalNAc production induced by icariin provided carbon and nitrogen sources through multiple metabolic pathways to support Akk growth. The generated GalNAc entered glycolysis to produce pyruvate, which subsequently fuels the TCA cycle for ATP generation, while its acetyl groups served as nitrogen sources for amino acid and nucleotide biosynthesis (Figure 5G,H). Notably, unlike other glycoside hydrolases, the catalytic activity of Amuc_0920 depends on NAD^+^ as a cofactor [42]. Metabolomic analysis of Akk revealed that the icariin group promoted a notable decrease in cofactor NAD^+^ levels, suggesting extensive consumption during substrate metabolism (Figure 6D). Meanwhile, the level of the NAD^+^ precursor NMN increased markedly, indicating that Akk may have activated a compensatory NAD^+^ biosynthetic pathway in response to this depletion (Figure S4B).

To further investigate the molecular mechanism by which icariin enhances the glycosidic bond cleavage activity of Amuc_0920 toward GalNAc, we employed AlphaFold3 to construct multiple complex systems containing icariin, Amuc_0920, NAD^+^, and GalNAc, followed by 100 ns molecular dynamics (MD) simulations. During the last 40 ns of the MD simulations, all complex systems (Amuc_0920‐NAD^+^, Amuc_0920‐NAD^+^‐GalNAc, Amuc_0920‐NAD^+^‐icariin, and Amuc_0920‐NAD^+^‐icariin‐GalNAc) exhibited stable root‐mean‐square deviation (RMSD) values around 0.2 nm, indicating that these systems had reached conformational equilibrium (Figure 6E). The root‐mean‐square fluctuation (RMSF) analysis revealed that the majority of residues in Amuc_0920 exhibited low flexibility across all complex systems, indicating high overall structural stability (Figure 6F). Meanwhile, the radius of gyration (Rg) of Amuc_0920 remained stable at approximately 2.18 nm in all systems, confirming that the protein maintained a compact and stable conformation throughout the MD simulations (Figure 6G). Hydrogen bond analysis (Hbnum) showed that during the last 40 ns of MD simulations, an average of 9.6 hydrogen bonds were formed between Amuc_0920‐NAD^+^ and icariin, indicating stable binding between icariin and Amuc_0920‐NAD^+^ (Figure 6H). Furthermore, while only 3.5 hydrogen bonds were detected between Amuc_0920‐NAD^+^ and the substrate GalNAc, the presence of icariin increased this interaction by 5.1 additional hydrogen bonds, suggesting that icariin enhanced the binding stability of GalNAc with Amuc_0920‐NAD^+^ (Figure 6H). Principal component analysis (PCA) revealed that the principal motion (PC1) of the Amuc_0920‐NAD^+^‐Icariin‐GalNAc complex accounted for the highest proportion of overall conformational changes, with the fastest curve decline, indicating more concentrated principal conformational fluctuations and greater structural stability in this bound state (Figure 6I). Free energy landscape (FEP) analysis further revealed that the addition of icariin to the Amuc_0920‐NAD^+^‐GalNAc system regulated the structural dynamics and stability of Amuc_0920 in different ligand‐bound states (Figure 6J). 3D interaction analysis found that when GalNAc binds alone, only a limited number of residues on Amuc_0920 participate in stabilizing the binding interface. However, in the presence of icariin, additional key residues (including GLU240, ARG244, TYR339) were observed surrounding GalNAc, forming a tightly packed binding interface (Figure 6K). MMGBSA analysis showed that the binding free energy between GalNAc and Amuc_0920‐NAD+ decreased significantly from −8.79 ± 3.50 to −23.82 ± 4.26 kcal/mol upon icariin binding, indicating a substantial enhancement in substrate affinity (Figure 6L,M). Binding free energy decomposition further demonstrated that icariin markedly increased the energetic contributions of several key residues (including TYR226, GLU240, ARG244, TYR256, TYR339, and HIS404) toward GalNAc binding, confirming stronger molecular interactions in the cooperative binding state (Figure 6M). Subsequently, we used the artificial substrate pNP‐GalNAc to examine the effect of icariin on the enzymatic activity of N‐acetylgalactosaminidase Amuc_0920 derived from Akk. The enzyme activity curves revealed that, compared with the vehicle group, the icariin‐treated group exhibited a significantly higher absorbance of pNP (p‐nitrophenol, the yellow hydrolysis product) at 405 nm, with statistically significant differences observed starting at 10 min (Figure 6N). Therefore, these results showed that icariin increased the hydrolytic activity of N‐acetylgalactosaminidase Amuc_0920 in Akk, which promoted enhanced mucin metabolism and bacterial growth.

### 2.7 Icariin‐Induced Akk Enrichment in the Gut Microbiota Modulated Immune Responses Leading to Tumor Suppression in LLC and B16 Mouse Models

**FIGURE 7:**
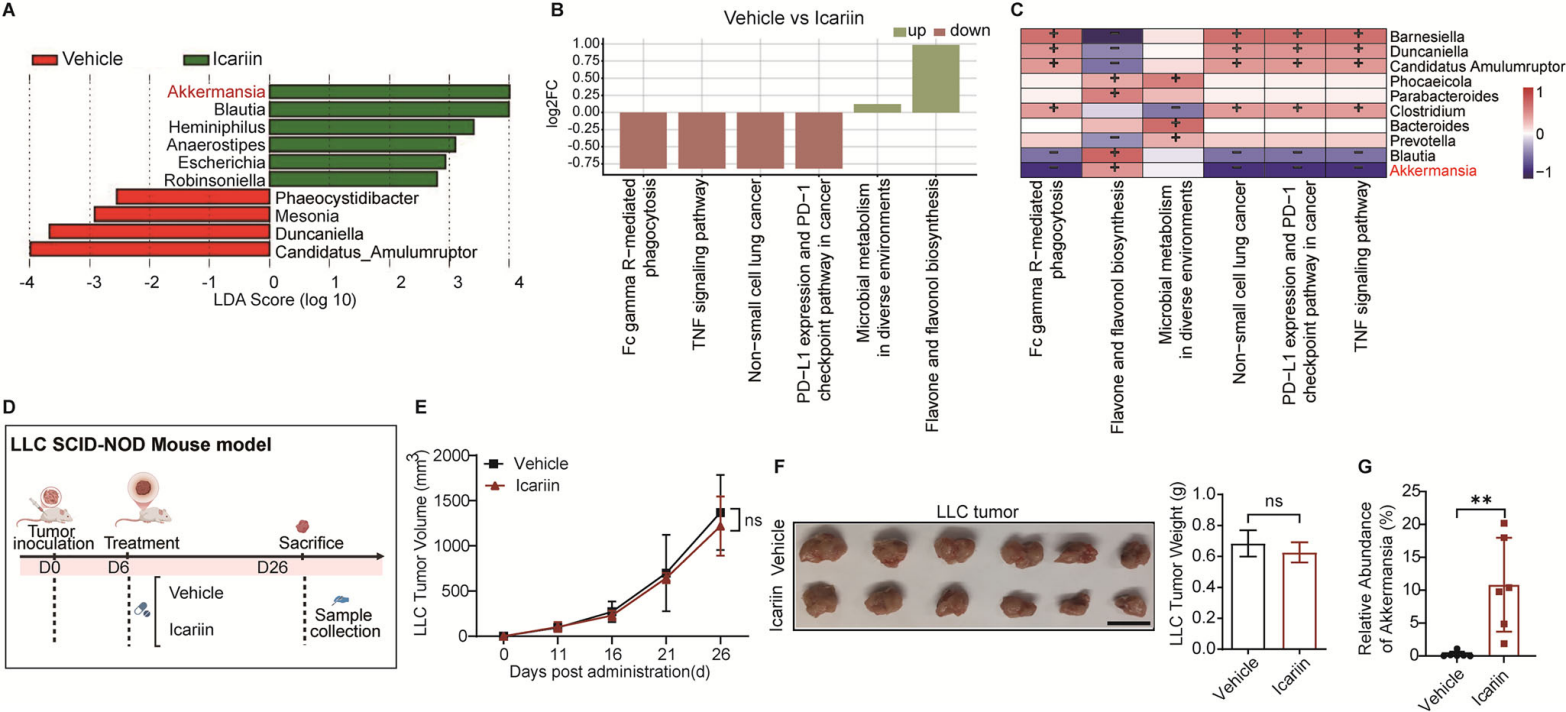
Gut microbial pathways modulated by icariin in the LLC mouse model by metagenomic analysis. (A) Linear discriminant analysis (LDA) with effect size measurement for significantly abundant taxa in LLC tumor‐bearing mice, with icariin‐enriched taxa (green, positive LDA scores) and vehicle‐enriched taxa (red, negative scores). (B) Bar plot for microbial KEGG pathways in LLC tumor‐bearing mice receiving with icariin or vehicle. (C) Correlation heatmap for microbial KEGG pathways and signature microbial taxa in LLC tumor‐bearing mice receiving with icariin or vehicle. (D) Experimental design of icariin treatment in LLC SCID‐NOD mouse model. (E) Tumor growth curve in LLC tumor‐bearing SCID‐NOD mice receiving with vehicle or icariin (70 mg/kg) (*n* = 6/group). (F) Tumor photographs and statistics of LLC tumor‐bearing SCID‐NOD mice receiving with vehicle or icariin (*n* = 6/group), scale bar: 1 cm. (G) The relative abundance of fecal Akk in LLC tumor‐bearing SCID‐NOD mice receiving with vehicle or icariin by qPCR analysis (*n* = 6/group). Two‐tailed unpaired t‐test: ^*^ *p* < 0.05, ^**^ *p* < 0.01.

Shotgun metagenomics serves as a powerful tool for elucidating the functional potential of microbial communities, extending beyond the taxonomic profiling offered by 16S rRNA sequencing. Metagenomic analysis of fecal samples from vehicle or icariin‐treated LLC mice was performed to elucidate the functional regulation of icariin‐induced Akk enrichment in the gut microbiota. Consistent with 16s RNAseq analysis, Akk was significantly enriched in the icariin group by LDA analysis (Figure 7A). Subsequently, functional annotation of metagenomic data was performed using the KEGG database. KEGG functional gene analysis revealed significant differences between the icariin and vehicle groups. Notably, pathways such as Flavone and flavonol biosynthesis and Microbial metabolism in diverse environments pathways were upregulated in the icariin group (Figure 7B). Correlation heatmap microbial KEGG showed a significant positive correlation between Akk and the flavone and flavonol biosynthesis pathway, suggesting Akk might be involved in the icariin biosynthesis (Figure 7C). In contrast, immune suppression pathways such as PD‐L1 expression and PD‐1 checkpoint pathway in cancer and TNF signaling pathway were downregulated in the icariin group (Figure 7B). Furthermore, the correlation heatmap showed that Akk is a potential negative correlation with these two pathways, indicating that higher levels of is associated with reduced expression or activity of these immunosuppressive pathways (Figure 7C). These findings demonstrated that Akk enriched by icariin exhibits crucial effects on the immune regulation pathways.

Emerging evidence suggests that Akk modulates cancer therapeutic outcomes by reshaping host immune responses [9, 43]. In Figure 3E–H, we observed that icariin administration increased Akk abundance in the gut microbiota of immunodeficient NOD/SCID mice. We next aim to determine whether the anti‐tumor efficacy mediated by icariin‐enriched Akk requires immune system involvement. Therefore, we administered icariin to LLC tumor‐bearing SCID‐NOD mice, and found that icariin failed to attenuate tumor growth compared to vehicle groups, as evidenced by the tumor volume curve and tumor weight analysis (Figure 7D–G). This loss of anti‐tumor activity was further confirmed in B16 tumor‐bearing SCID‐NOD mice, where icariin treatment showed nearly no inhibitory effects on tumor progression (Figure S5A–C). Consistent with our findings in SCID‐NOD mice, the anti‐tumor efficacy of icariin was also abolished in Rag1‐KO immunodeficient C57BL/6 tumor‐bearing mice, compared to immune competent C57BL/6 mice (Figure 1; Figure S6). These collective findings suggested that host immunity is essential for icariin's therapeutic effects.

### 2.8 Single‐Cell Sequencing Analysis Elucidated the Impact of Icariin‐Induced Akk Enrichment in the Gut Microbiota on the Tumor Microenvironment Within the LLC Mouse Model

To investigate the influence of icariin‐induced Akk enrichment in gut microbiota on TME, immune cells, particularly, single‐cell sequencing analysis on tumor samples collected from the FMT LLC mouse model in Figure 1K. Icariin FMT and vehicle FMT were used for treatment in the LLC mouse model to eliminate the possibility of direct influence from icariin within the tumor.

**FIGURE 8:**
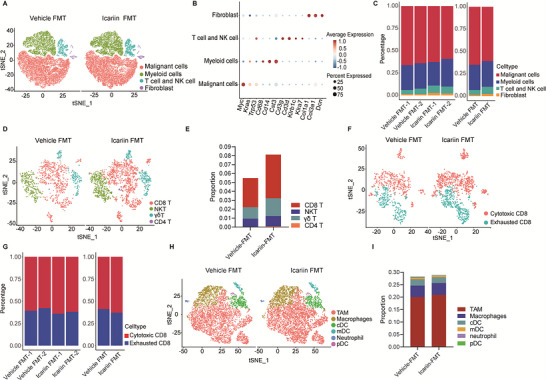
Single‐cell analysis delineated the transcriptomic landscape between icariin‐FMT treatment and vehicle‐FMT treatment in the LLC mouse model. (A) T‐distributed stochastic neighbor embedding (t‐SNE) analysis identifying 4 main cell types (malignant cells, myeloid cells, T and NK cells, and fibroblasts) in LLC tumor‐bearing mice receiving with icariin‐FMT or vehicle‐FMT (*n* = 2/group). (B) Feature Plot visualization of canonical cell‐type marker gene expression across annotated cell populations in LLC tumor‐bearing mice. (C) Histogram analysis showing the percentage of 4 main cell types (malignant cells, myeloid cells, T and NK cells, and fibroblasts) in LLC tumor‐bearing mice receiving with icariin‐FMT or vehicle‐FMT. (D) t‐SNE analysis of T cell and NK cell subpopulations in LLC tumor‐bearing mice receiving with icariin‐FMT or vehicle‐FMT. (E) Histogram analysis showing the proportion of T cells and NK cells in LLC tumor‐bearing mice receiving with icariin‐FMT or vehicle‐FMT. (F, G) t‐SNE (F) and histogram analysis (G) showing the percentage of CD8 T cell subpopulations in LLC tumor‐bearing mice receiving with icariin‐FMT or vehicle‐FMT. (H) t‐SNE analysis of Myeloid cells subpopulation in LLC tumor‐bearing mice receiving with icariin‐FMT or vehicle‐FMT. (I) Histogram analysis showing the proportion of Myeloid cells in LLC tumor‐bearing mice receiving with icariin‐FMT or vehicle‐FMT.

Using Cell Ranger (10X Genomics) and the Seurat package for stringent quality filtering, we generated a gene‐cell matrix with 46 603 cells (21 835 from vehicle FMT mice and 24 768 from icariin FMT mice) across 23 643 genes. Through t‐distributed stochastic neighbor embedding (t‐SNE) analyses, unsupervised clustering identified 15 unique cellular clusters (Figure S7A,B). According to their gene profiles and canonical markers, 4 main clusters were identified. Through matching cluster‐enriched genes to established cell type markers from previous studies, the 4 clusters were annotated as malignant cells (Mycs, Kras, and Trp53), myeloid cells (Cd14, Cd68, and Cst3), T cells and NK cells (Cd3g, Cd3d, Klrb1c, and Klra7), and fibroblast (Col1a1, Col3a1 and Dcn) (Figure 8A,B). The relative fractions of each cluster in the LLC tumor samples within two groups are shown in Figure 8C. Notably, the percentage of T cell and NK cell clusters in the icariin FMT group was specifically higher than that in the vehicle FMT group. Additionally, the icariin FMT group had fewer malignant cells compared to its vehicle counterpart. Although myeloid cells constituted the majority population of the immune cells, there was no notable difference in the proportions of the two groups (Figure 8C).

Re‐clustering analysis on 3208 cells of T cell and NK cell lineage revealed 4 clusters, including CD8+ T cells, CD4+ T cells, NK T cells, and γδT cells (Figure 8D). Each cluster was identified by known markers reported in previous studies, CD8+ T cells (Trbc2, Cd8a, Cd8b1, Cd3g), CD4+ T cells (Cd3e), NKT cells (Klrb1c, Klra7, Nkg7), and γδT cells (Rora, Trdc, Tcrg‐C1) (Figure S7C). Among the 4 clusters, CD8+ T cells contributed to the major population. Furthermore, the proportion of CD8+ T cells was significantly higher in the icariin FMT group compared to the vehicle FMT group (Figure 8E). Moreover, when 1,922 CD8+ T cells were further subclustered into cytotoxic and exhausted T cells by the FindAllMarkers function (Figure 8F; Figure S7D), the icariin FMT group showed a higher percentage of the cytotoxic CD8+ T cell subcluster and a lower percentage of the exhausted CD8+ T cell subcluster compared to the vehicle FMT groups (Figure 8G). Consistent with these findings, flow cytometry analysis in LLC tumor‐bearing mice further confirmed that icariin‐FMT significantly promoted the infiltration of CD8+ T cells into the tumor, with the proportion of CD8+ T cells among CD45+ cells being 34.8% in the icariin‐FMT group compared to 22.5% in the vehicle‐FMT group (Figure S8A). In addition, analysis of other immune cell subsets showed that icariin‐FMT also significantly increased the proportion of CD4+ T cells and NKT cells, while the proportions of γδT cells were elevated but did not reach statistical significance (Figure S8B–D). In contrast, re‐clustering analysis on 13 319 myeloid cells revealed a total of 6 clusters, including tumor‐associated macrophages (TAMs) (Fcna, Lyve1, Npl, Arg1, Tmem176b, C1qb), monocyte‐derived macrophages (Plac8), neutrophil (S100a8), mDC (Cacnb3), cDC1 (Plet1, Cd209a), and pDC (Ly6d) (Figure 8H; Figure S7E). However, no significant difference in percentage was observed for 6 clusters between the icariin FMT and vehicle FMT groups (Figure 8I).

In addition, we performed the differential gene expression analysis on malignant cells between the two groups. 38 up‐regulated differentially expressed genes (DEGs) were identified in the icariin FMT group by using the R package ‘limma’. GO enrichment analysis showed that the most enriched GO pathways are oxygen‐associated, including ‘response to hypoxia’, ‘response to decreased oxygen levels’, ‘haptoglobin hemoglobin complex’, ‘haptoglobin binding’, ‘antioxidant activity’, ‘oxygen binding’, ‘hemoglobin binding’, and ‘oxygen carrier activity’, suggesting that the icariin induced gut microbiota change may have the potential to impact the hypoxia condition in TME (Figure S7F).

### 2.9 Icariin‐Induced Akk Enrichment Contributed to the Suppression of Malignant Cells and the Activation of CD8+ T Cells in the TME

**FIGURE 9:**
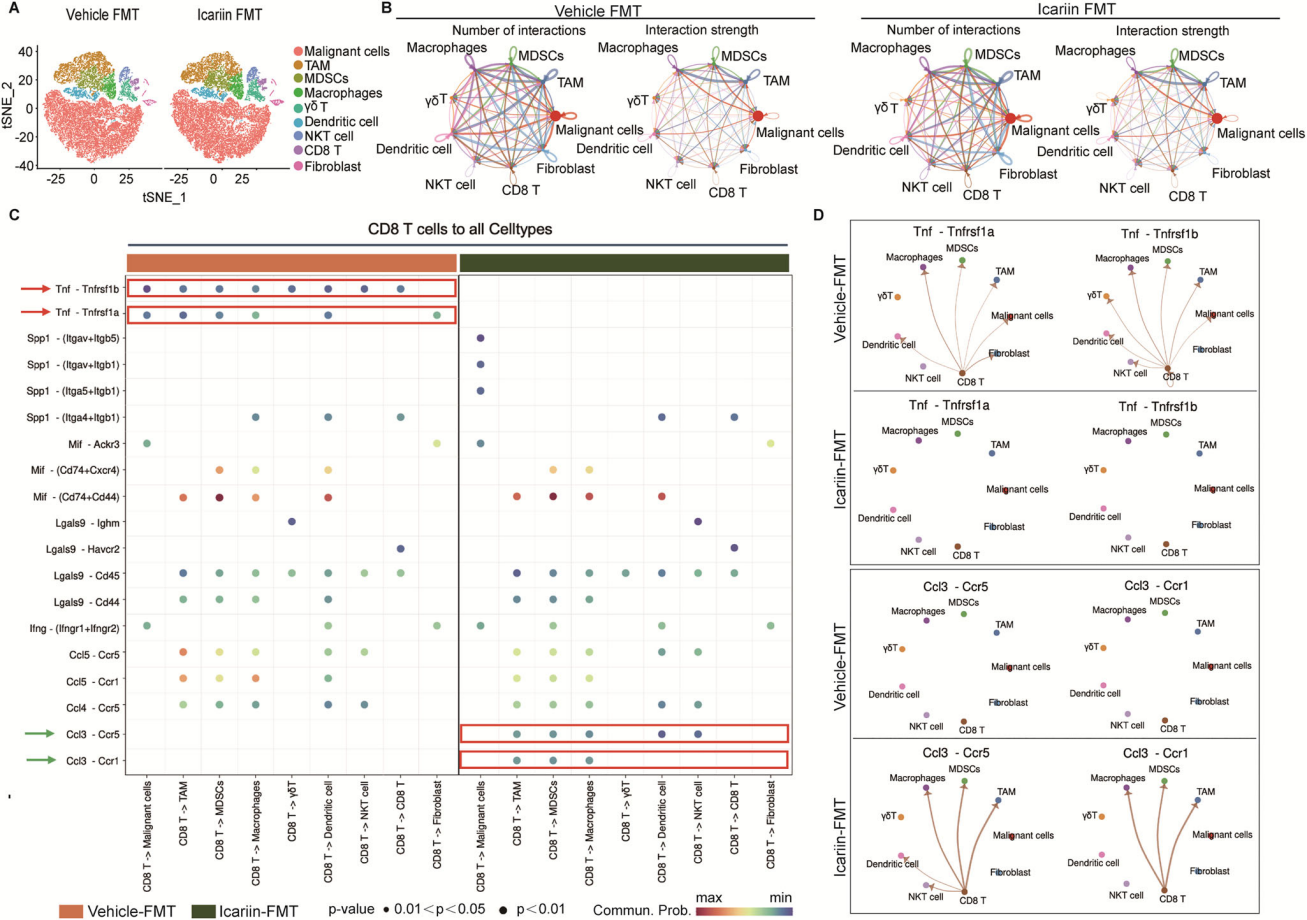
Icariin‐FMT reprogrammed intercellular crosstalk to activate CD8+ T cell‐mediated antitumor immunity. (A) T‐distributed stochastic neighbor embedding (t‐SNE) analysis identifying 9 main cell subtypes (malignant cells, TAM, MDSCs, Macrophages, γδT, Dendritic cell, NKT cell, CD8 T, and fibroblasts) in LLC tumor‐bearing mice receiving with icariin‐FMT or vehicle‐FMT (*n* = 2/group). (B) The overall information flow with different significances shows the number and strength of intercellular interactions in LLC tumor‐bearing mice receiving with icariin‐FMT or vehicle‐FMT. (C) Bubble chart showing the ligand‐receptor binding analysis of all pathways of CD8 T cells acting on other cell types between icariin‐FMT and vehicle‐FMT groups. (D) Intercellular interaction network analysis showing signaling pathways between CD8 cells and other cell types influenced by icariin‐FMT treatment.

As CD8+ T cells play a crucial role in tumor immunology and immunotherapy, with closely association with Akk enrichment in gut microbiota, we further applied intercellular interaction network analysis to explore how CD8+ T cells interact with malignant cells and other cell types in TME after icariin FMT treatment (Figure 9A,B). Ligand‐receptor (L‐R) analysis showed that the icariin FMT group greatly downregulated the TNF pathway activation between CD8+ T cells with other cell types, including malignant tumor cells (Figure 9C,D). The TNF pathway in CD8+ T cells has been previously reported to contribute to CD8+ T cell exhaustion and immune escape/suppression; the weakening of this signaling pathway within the tumor microenvironment would relief immune suppression and relieve immune activation [44, 45]. We also found that the signaling pathways of integrins such as Itgav+Itgb5, Itgav+Itgb1, and Itga5+Itgb1 between CD8+ T cells and malignant cells were specifically activated in the icariin FMT group (Figure 9C,D). Moreover, interaction between CD8+ T cells with other suppressive immune cells such as TAM and MDSCs, in the icariin FMT treatment group also showed upregulated Ccl3‐Ccr1 and Ccl3‐Ccr5 signaling (Figure 9C,D). Ccl3‐Ccr1 and Ccl3‐Ccr5 signaling are classic pathways that recruit and stimulate the activation and proliferation of immune cells for sustained immune surveillance and cytotoxic activity, subsequently targeting and destroying infected or cancer cells [46, 47]. This suggested that the icariin FMT treatment would convert the original suppressive immune environment to a more surveillant and active state, which is more favorable to achieve tumor elimination and inhibition.

### 2.10 Icariin‐Induced Akk Enrichment in Gut Microbiota Enhanced the Efficacy of PD‐1‐Based Immunotherapy in LLC and B16 Mouse Models

**FIGURE 10:**
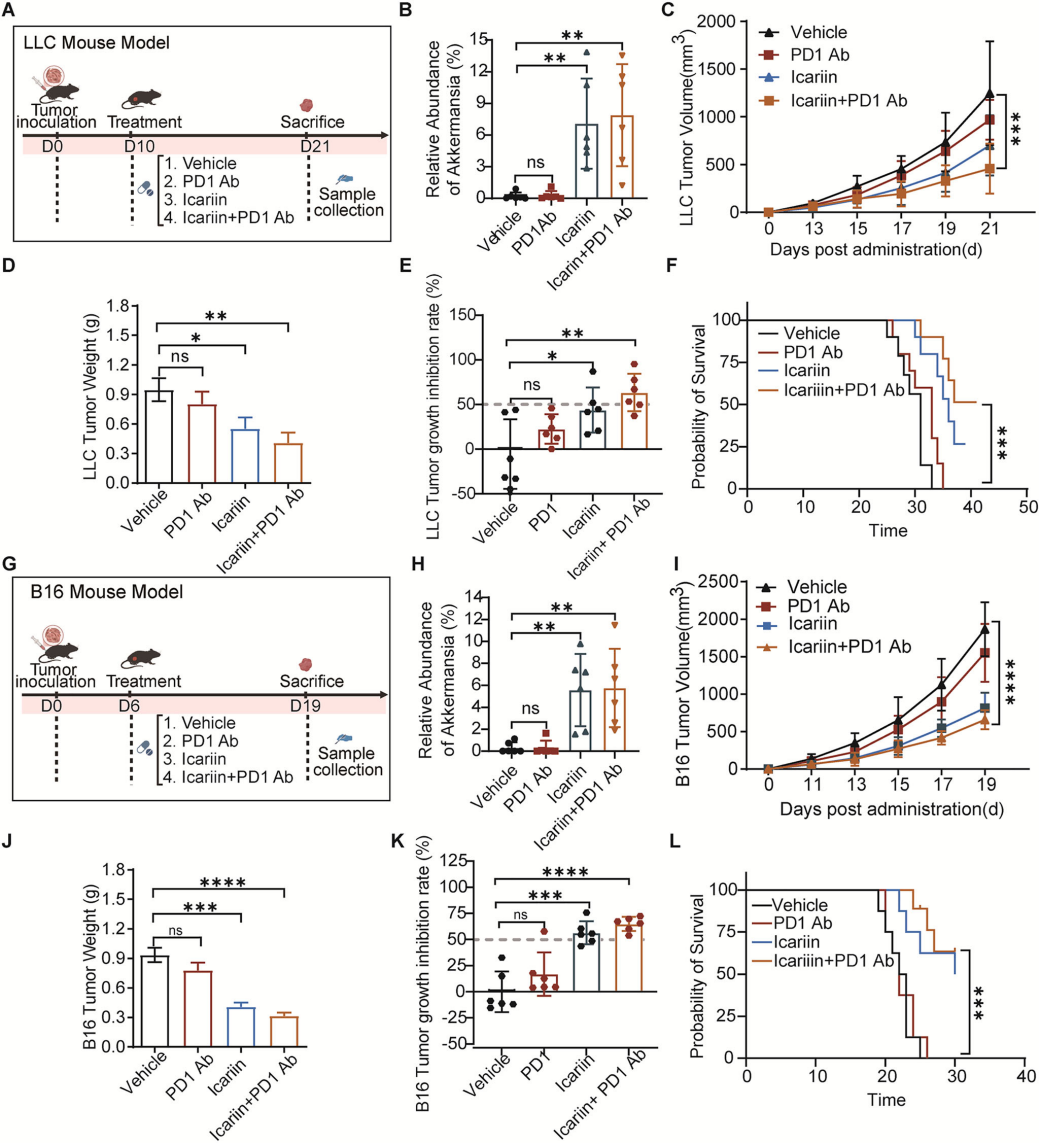
Icariin‐induced Akk enrichment in gut microbiota enhanced the efficacy of PD‐1‐based immunotherapy in LLC and B16 mouse models. (A) Experimental design for icariin combined with anti‐PD1 Ab treatment in the LLC mouse model. (B) The relative abundance of fecal Akk in LLC tumor‐bearing mice receiving vehicle, anti‐PD1 Ab (5 mg/kg), icariin (70 mg/kg), or a combination of icariin and anti‐PD1 Ab, respectively, by qPCR analysis (*n* = 6/group). (C) Tumor growth curve in LLC tumor‐bearing mice receiving vehicle, anti‐PD1 Ab, icariin, or a combination of icariin and anti‐PD1 Ab, respectively (*n* = 6/group). (D) Tumor weight in LLC tumor‐bearing mice receiving vehicle, anti‐PD1 Ab, icariin, or a combination of icariin and anti‐PD1 Ab, respectively. (E) Tumor growth inhibition rate in LLC tumor‐bearing mice receiving vehicle, anti‐PD1 Ab, icariin, or a combination of icariin and anti‐PD1 Ab, respectively. The gray line represents 50% Tumor growth inhibition rate. (F) Kaplan–Meier survival curve in LLC tumor‐bearing mice receiving vehicle, anti‐PD1 Ab, icariin, or a combination of icariin and anti‐PD1 Ab, respectively. (*n* = 6/group). (G) Experimental design for icariin combined with anti‐PD1 Ab treatment in the B16 mouse model. (H) The relative abundance of fecal Akk in B16 tumor‐bearing mice receiving vehicle, anti‐PD1 Ab (5 mg/kg), icariin (70 mg/kg), or a combination of icariin and anti‐PD1 Ab, respectively, by qPCR analysis (*n* = 6/ group). (I) Tumor growth curve in B16 tumor‐bearing mice receiving vehicle, anti‐PD1 Ab, icariin, or a combination of icariin and anti‐PD1 Ab, respectively (*n* = 6/ group). (J) Tumor weight in B16 tumor‐bearing mice receiving vehicle, anti‐PD1 Ab, icariin, or a combination of icariin and anti‐PD1 Ab, respectively. (K) Tumor growth inhibition rate in B16 tumor‐bearing mice receiving vehicle, anti‐PD1 Ab, icariin, or a combination of icariin and anti‐PD1 Ab, respectively. The gray line represents 50% Tumor growth inhibition rate. (L) Kaplan–Meier survival curve in B16 tumor‐bearing mice receiving vehicle, anti‐PD1 Ab, icariin, or a combination of icariin and anti‐PD1 Ab, respectively (*n* = 6/group). Two‐tailed unpaired t‐test and Kaplan–Meier analysis. ^*^ *p* < 0.05; ^**^ *p* < 0.01, ^***^ *p* < 0.001, ^****^ *p* < 0.0001.

Akk has emerged as a key predictive biomarker for anti‐PD1 antibody therapy efficacy [9]. Therefore, we evaluated the ability of icariin to enhance the response to anti‐PD1 Ab therapy through Akk enrichment in LLC and B16 mouse models. In the LLC mouse model, qPCR analysis revealed that the relative abundance of Akk in the fecal gut microbiota notably increased following treatment with icariin, either as a monotherapy or combined with anti‐PD1 Ab (Figure 10A,B). Subsequent tumor growth curve analysis showed that while anti‐PD1 Ab monotherapy slightly inhibited tumor progression, the combination therapy (icariin therapy combined with anti‐PD1 Ab therapy) resulted in significant tumor growth suppression (Figure 10C,D; Figure S9A). Notably, the 50% tumor inhibition rate reached ∼16.7% with anti‐PD1 Ab monotherapy but increased to ∼83.3% in the combination therapy (Figure 10E). Flow cytometry analysis found that both icariin alone and in combination with anti‐PD1 Ab significantly increased CD8+ T cell infiltration in tumors (Figure S10A). ELISA analysis further revealed that icariin alone and in combination with anti‐PD1 Ab increased the expression levels of granzyme B, a key effector molecule secreted by activated cytotoxic CD8+ T cells (Figure S10B). Furthermore, survival analysis confirmed that the combination therapy remarkably prolonged the overall survival in LLC tumor‐bearing mice (Figure 10F).

**FIGURE 11:**
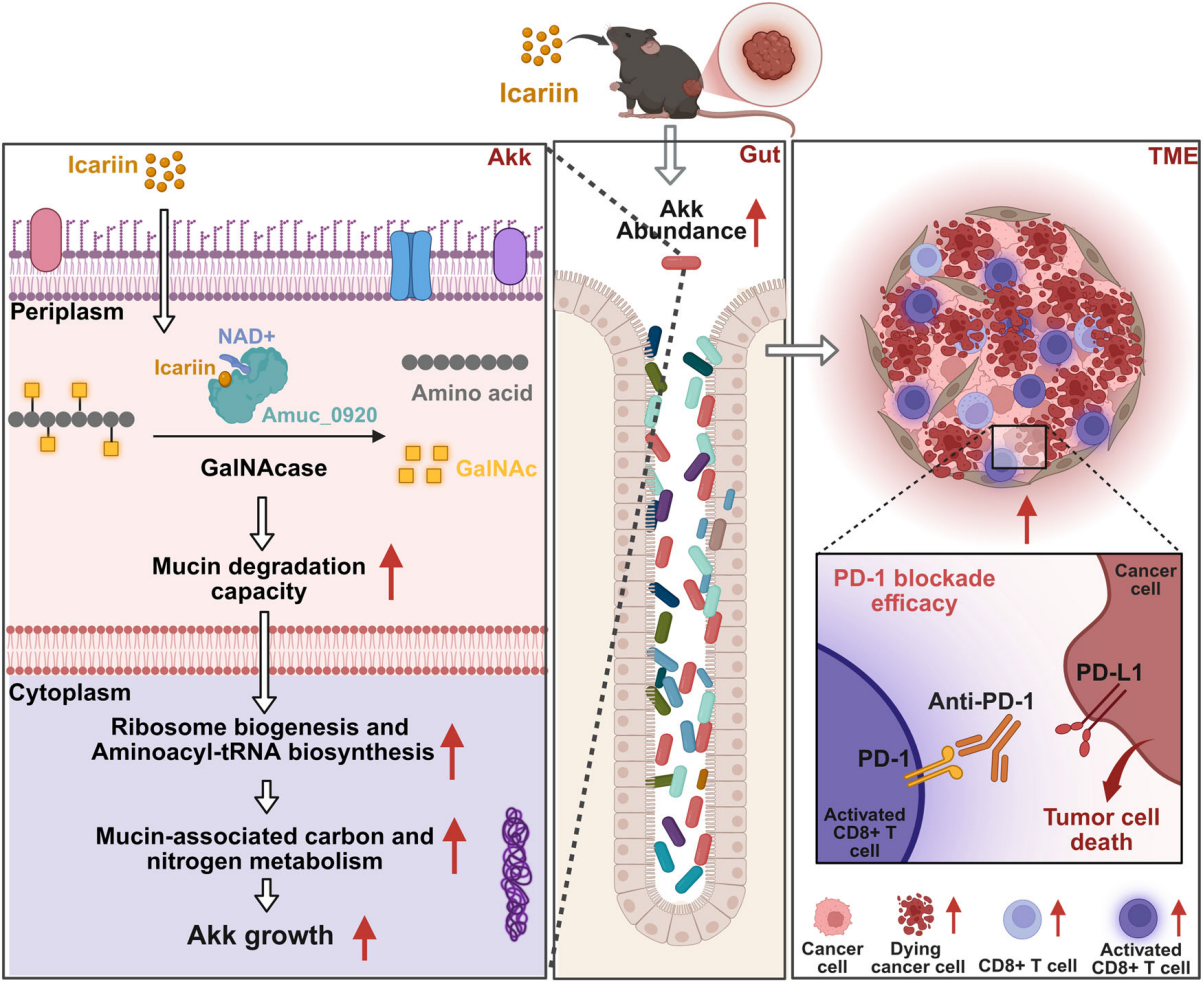
Icariin promoted the growth of Akk by enhancing the activity of N‐acetylgalactosaminidase (Amuc_0920), which enhanced mucin utilization and provided a favorable nutrient environment for bacterial growth. This icariin‐mediated enrichment of Akk further reshaped the tumor microenvironment and promoted CD8+ T cell infiltration, ultimately synergizing with PD‐1 blockade therapy to suppress tumor progression.

In the B16 mouse model, fecal abundance of Akk also exhibited a marked elevation in the combination therapy (icariin therapy combined with anti‐PD1 Ab therapy) compared to anti‐PD1 Ab monotherapy (Figure 10G,H). Moreover, the combination therapy was observed to strongly inhibit tumor growth, and increased the 50% tumor inhibition rate to 100% by tumor volume monitoring (Figure 10I–K; Figure S9B). Survival analysis further confirmed that the combination therapy significantly extended the survival period of B16 tumor‐bearing mice (Figure 10L). In summary, these results indicated that icariin enhanced the efficacy of PD‐1‐based immunotherapy in both LLC and B16 tumor models while enriching Akk abundance in the fecal gut microbiota (Figure 11).

## 3 Discussion

The gut microbiota, particularly Akk, has garnered significant attention for its potential role in cancer therapy due to its ability to enhance gut barrier integrity, modulate host immunity, and improve therapeutic responses to immunotherapy [10, 16]. However, the clinical application of Akk‐based interventions faces considerable challenges. First, achieving efficient and stable intestinal colonization remains difficult, as Akk's abundance is highly dependent on host mucin availability and competes with other commensals. Second, large‐scale production under strict anaerobic conditions is costly and prone to batch‐to‐batch variability, limiting reproducibility. Additionally, interindividual differences in gut microbiota composition and host immune status further influence Akk colonization efficacy, leading to inconsistent therapeutic outcomes [48].

In this context, icariin offers several advantages over direct supplementation of Akk as well as other Akk‐promoting agents, such as metformin or dietary prebiotics. (1) Icariin overcomes key limitations of probiotic‐based therapies by promoting superior Akk colonization. Specifically, icariin potentiated Akk's mucin‐degrading capability by stabilizing key substrate‐binding residues of the N‐acetylgalactosaminidase Amuc_0920, enabling more efficient mucin utilization (Figure 6). We further found that icariin treatment significantly increased the expression levels of the key mucin protein Mucin‐2 in the colonic epithelium of LLC tumor‐bearing mice, both at the protein and mRNA levels (Figure S11). These data suggest that icariin not only enhances the mucin‐degrading activity of Akk but also promotes mucin expression in the host colon. This dual effect may provide more abundant nutritional substrates for Akk colonization, thereby facilitating its proliferation in the gut. Concurrently, metabolomic analysis revealed that icariin promoted the production of propionic acid by Akk (Figure S12). Previous studies have indicated that propionic acid secreted by Akk can upregulate the expression of the tight junction proteins ZO‐1 and occludin, thereby enhancing the integrity of the intestinal epithelial barrier [49]. Our IHC and qPCR analyses also showed that icariin treatment increased the protein and mRNA expression levels of tight junction proteins ZO‐1 and Occludin (Figure S13). In addition, the bioactivity of icariin is not limited to the parent compound: we observed that its intestinal metabolite icaritin similarly enhances Akk abundance, whereas structurally related metabolites such as baohuoside and desmethylicaritin lack this effect. This metabolite‐specific activity suggests that particular structural features, such as specific hydroxylation patterns, may be essential for promoting Akk colonization [50]. These findings may help explain why icariin is uniquely effective at overcoming the colonization barriers that often limit the efficacy of direct probiotic supplementation. Moreover, our metabolomic analysis data revealed that Akk cultured with icariin in vitro promoted the bioconversion of icariin into its related metabolites, including icaritin and baohuoside (Figure S14). (2) Icariin exhibits selectivity and helps maintain microbiota balance, because it specifically enriched gut Akk abundance without significantly altering overall microbiota diversity. While metformin has been shown to increase gut Akk abundance in type 2 diabetes patients, its effects are non‐specific and may disrupt other beneficial gut microbes [51, 52]. Similarly, prebiotics like ursodeoxycholic acid and inulin can enrich gut Akk but also often lack selectivity and exhibit variable efficacy across individuals [53, 54, 55]. In contrast, icariin demonstrated high specificity for Akk abundance without altering overall microbial diversity, as evidenced by both in vitro and in vivo models. Notably, icariin administration consistently enriched Akk abundance regardless of the immune status of the mice, suggesting broad applicability across patient populations, including those with compromised immune systems. (3) Icariin demonstrates excellent clinical safety, as evidenced by its long history of use in traditional Chinese medicine and validation in modern clinical trials [56]. Clinical trials in healthy adults have shown that even at high doses (up to 1,680 mg/day), icariin is still well tolerated, with only mild and transient gastrointestinal discomfort observed at the highest doses [57]. Importantly, no adverse effects on cognition or mood have been reported, further supporting its suitability for therapeutic use [57]. The convenience of oral administration and low production costs make icariin a practical alternative to live biotherapeutics, which are often limited by stringent manufacturing requirements and complex logistics.

Icariin‐induced Akk enrichment exerts a notable immunomodulatory effect on the tumor‐immune microenvironment. Recent studies have reported that icariin suppressed key pro‐inflammatory cytokines such as TNF‐α, IL‐6, and IL‐1β, which leads to both immunoregulatory and anti‐inflammatory effects. These actions reduce inflammation and suggest significant potential for icariin in cancer immunotherapy applications [58]. We confirmed that the antitumor effect of icariin required immune system participation, as it failed to inhibit tumor growth in immunodeficient SCID‐NOD mice, despite a similarly pronounced Akk enrichment. Furthermore, both antibiotic (ABX) and fecal microbiota transplantation (FMT) models demonstrated that the antitumor activity of icariin was mediated by the gut microbiota. We further performed single‐cell RNA sequencing analysis to explore the immunoregulatory role of icariin‐modulated gut microbiota within the tumor microenvironment. Our single‐cell data revealed that icariin‐FMT treatment in recipient mice specifically increased the population of CD8+ T cells and enhanced the cytotoxic activity of these cells in LLC tumors. This observation is consistent with Zhao et al., who reported that administration of live Akk reversed the exhausted state of CD8+ T cells and enhanced the cytotoxic function of CD8+ T cells in the tumor microenvironment, although the overall proportion of CD8+ T cells in tumors of LLC‐bearing mice did not change [59]. Mechanistically, we found that icariin‐FMT treatment downregulated TNF‐mediated signaling between CD8+ T cells and malignant cells, which has been implicated in T cell exhaustion and immunotherapy resistance by ligand‐receptor interaction analysis. Additionally, icariin‐enriched Akk enhanced the recruitment of immunostimulatory myeloid cells by upregulating the CCL3‐CCR1/CCR5 axis, thereby remodeling the tumor microenvironment toward a more immunogenic and less suppressive state. Similarly, Zhao et al. demonstrated that live Akk administration modulated intercellular communication within the tumor microenvironment, notably by attenuating the CXCL3–PD‐L1 axis‐mediated crosstalk between CAFs and PD‐L1(+) neutrophils [59]. Furthermore, live Akk suppressed the interaction between PD‐L1(+) neutrophils and CD8+ T cells, consequently rescuing CD8+ T cell function and enhancing antitumor immunity response [59]. Taken together, these single‐cell analysis findings revealed that both icariin‐FMT treatment (selectively Akk enrichment) and direct live Akk administration regulated the activation and function of CD8+ T cells within the tumor microenvironment in LLC tumor‐bearing mice through intercellular networks. Notably, icariin‐FMT treatment exhibits a more direct and pronounced effect on promoting both the proportion and activation of CD8+ T cells.

Icariin represents a promising, safe, and effective targeted prebiotic for Akk, highlighting its potential for clinical application as an Akk modulator. While our in vitro fecal microbiota culture experiments demonstrate icariin's Akk‐enriching effects, we acknowledge that these findings require validation in clinical populations, particularly in disease‐specific cohorts such as non‐small cell lung cancer patients. Due to the absence of relevant patient data, it remains uncertain whether the modulation of Akk by icariin observed in this study translates into clinically relevant outcomes in humans. Future studies should include longitudinal monitoring of Akk abundance alongside icariin treatment outcomes in relevant patient populations to establish clinically meaningful associations. Future work should also focus on advancing the clinical translation of icariin. For example, effective patient stratification will be crucial to address inter‐individual variability in gut microbiota. Assessment of baseline fecal Akk abundance may help identify Akk‐responsive subgroups. In addition, host immune profiling could further refine patient selection and improve prediction of therapeutic responses. Moreover, based on the pronounced efficacy of combined icariin and PD‐1 blockade observed in our LLC and B16 murine models, the rational development of synergistic therapies is particularly important. Clinical trials are needed to identify optimal therapeutic strategies combining icariin with immune checkpoint inhibitors in tumors characterized by low levels of intestinal Akk, such as NSCLC [60]. To further evaluate the synergistic effect of icariin, we identified a donor with an Akk relative abundance of only 0.001% (where the general abundance range of Akk in the gut microbiota is 1%–3%) [61]. Fecal microbiota from this donor was transplanted into microbiota‐depleted mice, followed by inoculation with LLC or B16 tumor cells. As shown in Figures S15A,D, the introduction of icariin significantly enhanced the Akk colonization in mice after Akk supplementation. This confirms that Akk combined with icariin is much more effective than Akk alone in increasing fecal Akk abundance, especially in gut environments lacking Akk. In LLC and B16 tumor models, although the Akk+anti‐PD1 Ab group suppressed tumor growth, there was no significant difference compared to the vehicle group (Figure S15B,C,E,F). Notably, the extra addition of icariin (Akk+icariin+anti‐PD1 Ab group) showed significantly greater tumor inhibition than any other group (Figure S15B,C,E,F). In summary, in models lacking Akk, combined supplementation with Akk and icariin improved Akk colonization and enhanced the response to PD‐1 immunotherapy compared to Akk alone. These findings provide important preclinical relevance to support the therapeutic potential of a combined Akk probiotic and icariin regimen to achieve Akk‐related beneficial effects.

## 4 Conclusion

This study establishes the flavonoid icariin as a potent and selective prebiotic that could significantly increase the abundance of the beneficial gut bacterium Akk. We found that icariin promoted Akk enrichment in both murine and human fecal microbiota cultures. In vitro assays confirmed that icariin promoted Akk growth in a mucin‐dependent manner. Transcriptomic and metabolomic analyses revealed that icariin enhanced mucin‐associated metabolic pathways to support Akk growth. Mechanistically, icariin increased the enzymatic activity of N‐acetylgalactosaminidase Amuc_0920‐NAD^+^ through structural stabilization of key substrate‐binding residues for GalNAc, consequently enhancing mucin catabolism and utilization.

This Akk growth‐promoting mechanism forms the basis of icariin's remarkable in vivo efficacy. Icariin promoted the significant and selective enrichment of intestinal Akk in various mouse models, including both LLC and B16 tumor‐bearing mice as well as non‐tumor mice, regardless of whether the mice were immunocompetent or immunodeficient. Furthermore, the functional impact of icariin‐induced Akk enrichment is substantial. It enhanced CD8+ T cell activation within the tumor microenvironment, which reduces the TNFα‐mediated immune suppression pathway and upregulates the CCL3 signaling pathway. Ultimately, icariin treatment strongly synergized with anti‐PD‐1 immunotherapy, markedly enhancing the antitumor efficacy of PD‐1 blockade and prolonging the survival in both LLC and B16 mice models.

Icariin shows strong potential for clinical application as a well‐characterized and safe natural compound that overcomes clinical limitations of live Akk probiotics, including colonization instability and production complexity. With excellent oral bioavailability, low cost, and selective modulation of Akk, icariin stands out as an ideal candidate for clinical development. This work not only highlights icariin as an Akk‐targeted prebiotic for cancer immunotherapy, but also demonstrates its unique ability to enhance anti‐PD1 therapy efficacy by enriching gut Akk. Together, these findings offer a promising, stable, and potentially safer therapeutic strategy that effectively enriches Akk to enhance cancer immunotherapy outcomes and facilitate future clinical translation.

## 5 Methods

### 5.1 Cell Culture

The LLC Lewis lung carcinoma (ATCC CRL‐1642) and B16‐F10 melanoma (ATCC CRL‐6475) cell lines were purchased from the American Type Culture Collection (ATCC, USA). Cells were cultured in DMEM medium (Gibco, Cat. #12800017, USA) supplemented with 10% fetal bovine serum (FBS, Gibco, Cat. #A5670701, USA) and 1% penicillin‐streptomycin (100 U/mL penicillin, 100 µg/mL streptomycin), maintained at 37°C in a humidified incubator with 5% CO_2_/95% air atmosphere.

### 5.2 Bacterial Culture

Akkermansia muciniphila (ATCC BAA‐835) was purchased from the ATCC and cultured in pre‐reduced brain heart infusion broth (BHI, Solarbio, #LA0360) at 37°C under strictly anaerobic conditions with 85% N_2_, 10% CO_2_, 5% H_2_ (Bactron300 Anaerobic Chamber Glovebox, Shel Lab Inc., USA) supplemented with 0.25% (w/v) porcine gastric mucin (Sigma–Aldrich, Cat. #M2378) and 5 mg/L resazurin (Solarbio, Cat. #R8150, China). After 5 days of incubation (OD600 ≈ 0.5‐1.0), Akk was harvested by centrifugation at 5000 × g for 15 min at 4°C for subsequent experiments.

### 5.3 The Growth Curve of Akk In Vitro

Logarithmic‐phase Akk was inoculated into 0.25% mucin‐BHI medium supplemented with various concentrations of icariin (0, 50, 75, or 100 µm) for dose‐dependent analysis, or with 100 µm icariin or its metabolites for comparative analysis, respectively. The cultures were maintained under anaerobic conditions at 37°C for 72 h. The growth of Akk was assessed quantitatively by OD600 at set time points with a Multiskan SkyHigh Microplate Spectrophotometer (Fisher Scientific, USA).

### 5.4 The Colony Formation Assay of Akk In Vitro

Gradient concentrations of icariin (0, 50, 75, 100, or 200 µm) were added to 0.25% mucin‐BHI agar plates. 100 µL of logarithmic‐phase Akk was spread onto the icariin‐supplemented 0.25% mucin‐BHI agar plates, then cultured under anaerobic conditions at 37°C for 48 h. After incubation, the resulting colonies were photographed and quantified with ImageJ software.

### 5.5 The Growth of Akk in Fecal Gut Microbiota In Vitro

Fresh fecal samples were collected from healthy C57BL/6 mice and human volunteers. Fecal samples were mixed in sterile phosphate‐buffered saline (PBS, Gibco, Cat. # 10010023, USA) and homogenized using a TissueLyser LT (50 Hz, 3 min) (QIAGEN, Cat. #85600, Germany). The homogenates were then filtered through a 70 µm stainless steel mesh to remove particulate impurities. The filtered mixture was centrifuged at 5000 × g for 10 min at 4°C. The fecal microbial cells were resuspended in 0.25% mucin BHI medium supplemented with 100 µm icariin or its metabolites (icaritin, baohuoside, Desmethylicaritin) for comparative analysis. The cultures were incubated anaerobically at 37°C for 72 h. After incubation, fecal microbial cells were collected, and the growth of Akk was assessed by qPCR.

### 5.6 qPCR Analysis for Akk Abundance

Total DNA was extracted from fecal samples using PureLink Microbiome DNA Purification Kit (Fisher Scientific, Cat. #A29790, USA). Primers targeting the 16S rRNA gene (Forward: GTGCCAGCMGCCGCGGTAA; Reverse: GGACTACHVGGGTWTCTAAT) and Akk (Forward: AGAGGTCTCAAGCGTTGTTCCGAA; Reverse: TTTCGCTCCCCTGGCCTTCGTGC) were synthesized by Shanghai Integrated Biotech Solutions Co., Ltd (China). qPCR was conducted on a Real‐Time PCR System (Applied Biosystems, Cat. #4453551, USA). Relative abundance of Akk was calculated using the 2^−ΔCt^ method, normalized to the universal bacterial 16S rRNA signal.

### 5.7 Analysis of pNP‐GalNAc Enzyme Activity

To measure N‐acetylgalactosaminidase Amuc_0920 enzyme activity, 25 µL of Akk treated with icariin or vehicle was mixed with 25 µL of 10 mm 4‐Nitrophenyl‐N‐acetylgalactosaminidase (pNP‐GalNAc, MCE, Cat. #HY‐W015996, USA) substrate and 150 µL of McIlvaine buffer (pH 5.0), then incubated at 37°C for 0, 10, 20, 40 min. The reaction was terminated by the addition of 100 µL of 0.5 m NaCO_3_ solution. The absorbance of the yellow pNP product was measured at 405 nm using a Multiskan SkyHigh microplate spectrophotometer to determine the amount of GalNAc released.

### 5.8 Molecular Dynamics Simulation

The initial protein structure used for the simulations was predicted by AlphaFold3 [62]. All molecular dynamics (MD) simulations were carried out using the GROMACS 2023.4 software with the Amber14SB force field [63]. The systems were solvated in a cubic box of TIP3P water molecules with a minimum buffer distance of 0.8 nm. Na^+^ and Cl^−^ ions were added to ensure system neutrality and an ionic strength of 0.15 m. Energy minimization was performed using the conjugate gradient method until the maximum force (Fmax) was less than 100 kJ/mol/nm. Subsequently, a 100 ps equilibration with position restraints was carried out at 298.15 K and 1 bar, applying V‐rescale temperature coupling and Berendsen pressure coupling. This step was followed by a 100 ns production run under identical conditions, but without position restraints. Following the simulations, trajectory analysis methods such as RMSD were utilized, and the binding free energy during the equilibrium phase was estimated using the MM‐GBSA approach with gmx_MMPBSA [64].

### 5.9 Prokaryotic Transcriptome Sequencing

Total RNA was isolated from Akk samples treated with either vehicle or icariin using the TRIzol reagent. A NanoDrop 2000 spectrophotometer (Thermo Scientific, Cat. #ND2000LAPTOP, USA) was utilized to determine the purity of the extracted RNA, ensuring the 260/280 absorbance ratio ranged from 1.8 to 2.0. The RNA integrity was subsequently assessed by agarose gel electrophoresis. After confirming RNA quality, ribosomal RNA was removed, and strand‐specific libraries were prepared using the NEBNext Ultra II Directional RNA Library Prep Kit for Illumina, with dual‐indexed adapters and size selection for inserts of 350 ± 50 bp. Qualified libraries were sequenced in paired‐end 150 bp (PE150) mode on the Illumina NovaSeq 6000 platform (Tsingke Biotechnology Co., Ltd., China) for downstream analysis.

### 5.10 LC‐QqQ‐MS Analysis of Mucin Degradation (N‐acetylgalactosamine, GalNAc)

Akk cultures treated with icariin or vehicle were centrifuged at 5000 × g for 10 min at 4°C to separate bacterial cells from the supernatant containing mucin degradation products. The supernatant was then treated with an equal volume of 80% methanol (1:1, v/v) to precipitate proteins, followed by incubation on ice for 30 min and centrifugation at 12 000 × g for 15 min. The mucin degradation (N‐acetylgalactosamine, GalNAc) in the supernatant was detected using an Agilent 6460 Triple Quadrupole LC‐QqQ‐MS system (Agilent Technologies, Cat. #G6460C, USA).

### 5.11 Untargeted Metabolomics Analysis of Akk

#### 5.11.1 Metabolite Extraction

Akk treated with either icariin or vehicle were collected by centrifugation (1000 × g, 30 min, 4°C), washed twice with pre‐cooled PBS, and rapidly frozen in liquid nitrogen. The cell pellets were subsequently sent to Qingke Biotechnology Co., Ltd. for untargeted metabolomics analysis. Briefly, the bacterial pellets (approximately 1 × 10^7^ cells) were mixed with 1000 µL of pre‐cooled extraction solution (methanol:acetonitrile:water = 2:2:1, v/v, containing deuterated internal standards). The mixture was vortexed for 30 s, frozen in liquid nitrogen for 1 min, and then thawed at room temperature. This freeze–thaw cycle was repeated three times. The samples were then sonicated in an ice‐water bath for 10 min and incubated at −40°C for 1 h to precipitate proteins. After centrifugation at 12 000 rpm for 15 min at 4°C, the supernatant was then used for LC‐MS analysis. To assess the stability of the analytical system, quality control (QC) samples were generated by combining equal aliquots from the supernatants of all samples.

#### 5.11.2 LC‐MS/MS Analysis

Untargeted metabolomics was conducted on a Vanquish UHPLC system (Thermo Fisher Scientific) coupled to an Orbitrap Exploris 120 mass spectrometer (Thermo Fisher Scientific). Polar metabolites were separated using a Waters ACQUITY UPLC BEH Amide column (2.1 mm × 50 mm, 1.7 µm; Waters, USA) maintained at 40°C. The mobile phase consisted of 25 mmol/L ammonium acetate and 25 mmol/L ammonium hydroxide in water (pH 9.75) as phase A, and acetonitrile as phase B. The elution gradient was: 0–1 min, 95% B; 1–14 min, 95%–65% B; 14–16 min, 65%–40% B; 16–18 min, 40% B; 18–18.1 min, 40%–95% B; and 18.1–23 min, 95% B. Flow rate was set to 0.5 mL/min, autosampler temperature to 4°C, and injection volume to 2 µL. The mass spectrometer alternated between positive and negative ionization modes. ESI source conditions included a sheath gas flow of 50 Arb, auxiliary gas flow of 15 Arb, capillary temperature at 320°C, and spray voltage of 3.8 kV (positive) or −3.4 kV (negative). For data acquisition, the full MS scan resolution was 60 000, and MS/MS resolution was set at 15,000 with stepped collision energies of 20, 30, and 40 eV.

#### 5.11.3 Data Analysis and Metabolite Annotation

The raw data files were converted to mzXML format using ProteoWizard (version 3.0.24054) and then analyzed using a custom R‐based workflow, which enabled peak detection, alignment, and integration. Metabolite identification was achieved by matching the precursor ion m/z (MS1), MS2 spectra, and retention time to a local standards database. Identified metabolites were categorized according to the Metabolomics Standards Initiative: Level 1 (MS1, MS2, and retention time matched with authentic standards), Level 2 (MS1 and MS2 matched with public databases), Level 3 (putatively annotated compounds), and Level 4 (unknown compounds). Statistical analysis was carried out using SIMCA software (version 18.0.1, Sartorius Stedim Data Analytics AB, Sweden). Metabolites showing significant differences were identified with a variable importance in projection (VIP) score greater than 1.0 from the OPLS‐DA model and a p‐value below 0.05 as determined by Student's t‐test.

#### 5.11.4 Animal Handling

Male C57BL/6J, immunodeficient SCID‐NOD, and Rag1‐KO immunodeficient mice (B6;129S‐Rag1^tm1(loxP‐EGFP‐PolyA‐loxP‐Neo‐loxP)^Smoc), aged 6–8 weeks, were obtained from Shanghai Model Organisms Center, Inc. (Shanghai, China) and housed in the animal facility at Hong Kong Baptist University. Animals were kept under standardized environmental conditions (22 ± 1°C, 55 ± 10% relative humidity, 12‐h light/dark cycle) with free access to autoclaved feed and reverse osmosis water. After a minimum 7‐day acclimatization period, experiments were initiated. All procedures were approved by the Hong Kong Baptist University Animal Experimentation Ethics Committee and conducted in accordance with institutional animal welfare guidelines.

#### 5.11.5 Subcutaneous Allograft Model for LLC or B16‐F10

Male mice were anesthetized with 3% isoflurane and placed in lateral recumbency. The right hind limb was shaved with electric clippers and disinfected with 75% ethanol. A suspension containing 5 × 10^5^ B16 cells or 1 × 10^6^ LLC cells in 100 µL sterile PBS was loaded into a 1 mL insulin syringe. The needle was inserted subcutaneously at a 45° angle into the prepared site and advanced horizontally for 1 cm to create a tissue pocket. The cell suspension was then slowly injected to minimize leakage. After injection, gentle pressure was applied to the site with a sterile cotton swab to ensure even distribution of the cells.

#### 5.11.6 Icariin Treatment and Tumor Evaluation

Tumor‐bearing mice were stratified into the following groups: vehicle control group (oral administration of corn oil, 0.2 mL/day), icariin monotherapy group (70 mg/kg/day icariin in corn oil, oral gavage), anti‐PD‐1 antibody monotherapy group (5 mg/kg anti‐PD‐1 antibody, intraperitoneal injection twice weekly), and combination therapy group (icariin 70 mg/kg combined with anti‐PD‐1 antibody 5 mg/kg). Body weight and tumor volume were measured using a calibrated digital caliper (Mitutoyo, ± 0.01 mm). Tumor volume (V) was calculated as V = (L × W^2^) / 2, where L and W represent the longest and shortest orthogonal diameters, respectively. Survival analysis was performed from the day of tumor inoculation, with endpoints defined as tumor volume exceeding 2,000 mm^3^ or 20% body weight loss. Kaplan–Meier curves were generated, and statistical significance was determined using Kaplan–Meier analysis in GraphPad Prism v9.0.

#### 5.11.7 Antibiotic (ABX) Treatment

According to the previously described [65, 66], with minor modification, tumor‐bearing mice received a one‐week treatment with a broad‐spectrum antibiotic cocktail (ABX, 0.5 g/L vancomycin, 1 g/L neomycin sulfate, 1 g/L metronidazole, and 1 g/L ampicillin) in sterile drinking water to eliminate the gut microbiota. The antibiotic‐containing water was replaced every day to ensure consistent efficacy. Successful depletion of the gut microbiota was verified by qPCR assays targeting the 16S rRNA gene.

#### 5.11.8 Fecal Microbiota Transplantation (FMT) Experiment

Fecal samples were collected from tumor‐bearing mice treated with vehicle or icariin and immediately processed as follows: After suspension in PBS at 30 mg/mL, the samples were homogenized using a TissueLyser LT (50 Hz, 3 min) (QIAGEN, Cat. #85600, Germany). The homogenate was then filtered through a 70 µm stainless‐steel mesh to remove particulate debris. The filtered suspension was centrifuged at 5000 × g for 10 min at 4°C. The resulting bacterial fraction was resuspended in sterile PBS for transplantation. Recipient tumor‐bearing mice were first subjected to a 1‐week broad‐spectrum antibiotic pretreatment (vancomycin 0.5 g/L, ampicillin 1 g/L, neomycin 1 g/L, 1 g/L ampicillin) to deplete endogenous gut microbiota. Subsequently, recipient mice received oral gavage (200 µL) of fecal suspension once daily until the experimental endpoint to ensure sustained microbial engraftment and gut microbiota modulation.

Therapeutic efficacy of combined icariin, Akk supplementation, and anti‐PD‐1 antibody in LLC/B16 tumor‐bearing mice with a lack of Akk. Mice were given sterile water containing a broad‐spectrum antibiotic cocktail, which was replaced daily for one week, to establish gut microbiota‐deficient mice. Fecal microbiota transplantation (FMT) was subsequently performed using donor feces that lacked Akk, with a relative abundance of only 0.001% in the gut microbiota. Donor fecal samples were homogenized, filtered, centrifuged, and resuspended in PBS. Mice were gavaged with 200 µL daily for three days to establish the model, and FMT was repeated weekly to maintain the microbiota composition. Once the model was established, mice were subcutaneously inoculated with LLC and B16 tumor cells and randomly divided into five groups (*n* = 6/group): (1) vehicle control group; (2) Akk supplementation group (1 × 10^8^ CFU/mouse/day by gavage); (3) Akk+anti‐PD‐1 antibody group (anti‐PD‐1 antibody, intraperitoneal injection, 5 mg/kg, twice per week); (4) Akk+icariin group (icariin, 70 mg/kg/day by gavage); (5) combination treatment group (Akk+icariin+anti‐PD‐1 antibody). All treatments were administered until the experimental endpoint. Tumor volume was measured every three days throughout the experiment. At the end of the treatment period, fecal samples were collected for analysis of Akk abundance, and tumors were harvested for imaging, weighing, and further analysis.

#### 5.11.9 Flow Cytometric Analysis of Immune Cell Subsets in LLC Tumor Tissues

Tumor tissues from LLC‐bearing mice were collected and washed with ice‐cold PBS (Gibco, USA) to remove fat and necrotic areas. They were minced and digested at 37°C in RPMI‐1640 medium (Gibco, USA) with collagenase I (1 mg/mL, Sigma–Aldrich, USA) and DNase I (0.1 mg/mL, Sigma–Aldrich, USA) for 30 min, with intermittent pipetting. After digestion, cells were filtered through a 70 µm cell strainer (Corning, USA), and red blood cells were lysed with ACK buffer (Gibco, USA). Cells were centrifuged at 500 × g for 5 min, resuspended in PBS, and counted. A total of 1 × 10^6^ cells were washed with PBS containing 2% FBS (Gibco, USA) and resuspended in flow cytometry buffer. Fc receptor blocking was conducted at 4°C for 15 min using the mouse Fc blocker (abs9477, Abclonal, China). Cells were stained with different combinations of the following antibodies: ABflo 488‐anti‐mouse CD45 (A23707, Abclonal, China), APC‐anti‐mouse CD3 (FMA003‐01, Suzhou 4A Biotech, China), PE‐anti‐mouse CD8 (FMP008‐02, Suzhou 4A Biotech, China), PE‐anti‐mouse CD4 (A25933, Abclonal, China), PE‐anti‐mouse TCRγδ (FMPTCRgd‐01, Suzhou 4A Biotech, China), and PE‐anti‐mouse NK1.1 (A24107, Abclonal, China), followed by incubation for 90 min at 4°C in the dark. After washing three times with PBS, cells were fixed with 1% PFA (Sigma‐Aldrich, USA) for 5 min. Samples were analyzed on a BD FACSDiscover S8 flow cytometer (BD Biosciences, USA). Data were analyzed using FlowJo to identify CD8+ T cells (CD45+CD3+CD8+), CD4+ T cells (CD45+CD3+CD4+), NKT cells (CD45+CD3+NK1.1+), and γδ T cells (CD45+CD3+TCRγδ+), and to calculate their proportions among CD45+ immune cells. All procedures were performed at low temperature and protected from light, with appropriate controls to ensure data reliability.

#### 5.11.10 Immunohistochemical Staining of Colon Tissue

Colon tissues were fixed in 4% paraformaldehyde, embedded in paraffin, and sectioned. Tissue sections were deparaffinized with xylene, rehydrated through a graded ethanol series, and underwent antigen retrieval using citrate buffer in a microwave. Endogenous peroxidase was inactivated by treating the sections with 3% methanol‐hydrogen peroxide for 25 min at room temperature in the dark. Subsequently, nonspecific binding was blocked with 3% BSA for 30 min at room temperature. Sections were incubated overnight at 4°C with the following primary antibodies: Anti‐MUC2 Rabbit pAb (GB11344, Servicebio, China), Anti‐ZO1 Rabbit pAb (GB115686, Servicebio, China), or Anti‐Occludin Mouse pAb (GB15149, Servicebio, China). After washing, the sections were incubated with HRP‐conjugated secondary antibodies for 1 h at room temperature. Positive signals were developed using DAB, after which slides were counterstained with hematoxylin, dehydrated in graded ethanol, cleared with xylene, and mounted using neutral balsam. Images were acquired using a Nikon E100 light microscope (Nikon Instruments, China).

### 5.12 Fecal Microbiota DNA Extraction, 16S rRNA Gene Sequencing, and Bioinformatics Analysis

#### 5.12.1 Fecal DNA Extraction and 16S rRNA Gene Sequencing

Genomic DNA was extracted from fecal samples collected from tumor‐bearing mice using the TIANamp Stool DNA Kit (TIANGEN Biotech, China), following the manufacturer's protocol. DNA quality and integrity were assessed by agarose gel electrophoresis, and concentration and purity were determined with a NanoDrop 2000 spectrophotometer, with qualified samples defined as having a 260/280 ratio ≥1.8 and 260/230 ≥2.0. The V3‐V4 regions of the bacterial 16S rRNA gene were amplified by PCR using universal primers 341F (5’‐CCTACGGGNGGCWGCAG‐3’) and 806R (5’‐GACTACHVGGGTATCTAATCC‐3’). The resulting amplicons were subjected to paired‐end sequencing on the Illumina NovaSeq PE250 platform (Magigene Biotechnology Co., China).

#### 5.12.2 Bioinformatics Analysis

Raw sequencing data were processed by merging paired‐end reads through overlapping regions using Pandaseq (v2.11). Quality filtering was performed on the RealBio analysis platform to remove sequences with average quality scores less than 30 (Q30), sequences with more than one ambiguous base (N), or lengths outside 250–500 nt, retaining high‐quality reads. High‐quality sequences were clustered into operational taxonomic units (OTUs) at 97% similarity using USEARCH (v7.0.1090), and singleton OTUs were discarded to minimize noise. OTU representative sequences were taxonomically assigned using the RDP Classifier (confidence threshold of 0.8) with the RDP 16S rRNA database (Release 11.5). OTU statistics and annotation rates for each sample are also reported. For diversity analyses, the number of reads per sample was rarefied to 24,061 (the smallest sample size). Alpha diversity (species richness and evenness) was evaluated using the Chao1, Shannon, and Simpson indices. Beta diversity was assessed by weighted and unweighted UniFrac distances, visualized via PCoA. Statistical significance of differences between groups was tested by permutational multivariate analysis of variance (PERMANOVA, 999 permutations). Additionally, LEfSe was used to identify biomarkers with statistical differences, with the LDA score threshold set at least 3.

#### 5.12.3 Metagenomics Sequencing and Data Analysis

Fecal genomic DNA was extracted from LLC tumor‐bearing mice treated with vehicle or icariin. DNA quality was determined by agarose gel electrophoresis and quantification with a NanoDrop 2000 Spectrophotometer. After constructing paired‐end libraries, sequencing was performed using the Illumina NovaSeq 6000 platform at Shenzhen Microscience Union Co., Ltd. (China). The bioinformatics pipeline included rigorous host DNA depletion through competitive alignment against the mouse reference genome, followed by microbial taxonomic profiling using the NCBI RefSeq database to achieve species‐level resolution in metagenomic characterization.

#### 5.12.4 Pre‐Processing of scRNA‐Seq Data

In R software (version 4.4.0), the Seurat package (version 4; http://satijalab.org/seurat/) was used to process the raw output data. Cells expressing fewer than 300 genes out of the total expressed genes were filtered out. 46,603 filtered cells in all were used for the bioinformatic analysis that follows.

#### 5.12.5 Data Integration and the Dimensionality Reduction of scRNA‐Seq

The Read10× function was applied to import gene expression data into Seurat objects for each sample. The 3000 most variable genes were identified from the normalized expression matrices and subsequently scaled. PCA was carried out utilizing these highly variable genes.

#### 5.12.6 Clustering and Cell Type Annotation of scRNA‐Seq

Cell clustering was performed using the Seurat “FindClusters” function based on a shared nearest neighbor graph, with clusters visualized by t‐SNE. Sub‐clustering analyses of selected groups followed the same pipeline, including dimensionality reduction and variable gene identification. Differentially expressed genes were identified using “FindAllMarkers” and, together with known marker genes from the literature, were used to annotate cell types. Details of the biomarkers used for annotation are provided.

#### 5.12.7 Cell–Cell Communication Network Inference of scRNA‐Seq

To investigate interactions between tumor cells and various cell types within the TME, we utilized the R package ‘CellChat’ (version 1.6.1). Our approach focuses on the ‘Secreted Signaling’ category from the ligand‐receptor pair database.

#### 5.12.8 Statistical Analysis

Statistical analyses were conducted using GraphPad Prism 9.5 (GraphPad Software, USA). Data with normal distribution are expressed as mean ± standard error of the mean (SEM). Based on data distribution and variance, group comparisons were performed using a two‐tailed unpaired t‐test for continuous variables and Kaplan–Meier analysis for survival data. Statistical significance was defined as *p* < 0.05.

## Author Contributions

F.L. is responsible for conceptualization, writing – original draft, writing editing, supervision, project administration, and funding acquisition. A.L. contributed to conceptualization, supervision, and funding acquisition. S.Q. performed writing – original draft, writing editing, methodology, software, validation, formal analysis, investigation, and visualization. L.Y. carried out writing the original draft, writing editing, methodology, software, validation, and formal analysis. H.H. and Q.D. were involved in methodology, software, validation, and writing and editing. F.D. contributed to writing, editing, methodology, software, and validation. Z.C. performed methodology, software, and validation. J.Z. participated in methodology and writing editing. Y.H. performed the methodology. M.L. worked on writing the original draft. J.X. and C.W. provided supervision.

## Conflicts of Interest

The authors declare no conflicts of interest.

## Supporting information




**Supporting File**: advs75414‐sup‐0001‐SuppMat.docx.

## Data Availability

The data that support the findings of this study are available in the supplementary material of this article.
