# Supplementary material for: Icariin Enhances the Enzymatic Activity of N‐acetylgalactosaminidase to Augment Akkermansia Abundance in Gut Microbiota for Improved PD‐1 Blockade Efficacy in Tumor Suppression
Source: Adv Sci (Weinh). 2026 Apr 22;13(40):e19942. doi: 10.1002/advs.202519942 (PMC13335675; doi:10.1002/advs.202519942)
Supplement: Supplementary file 1 — Supporting File: advs75414‐sup‐0001‐SuppMat.docx. [file ADVS-13-e19942-s001.docx]

**Supporting information**

**Icariin enhances the enzymatic activity of N-acetylgalactosaminidase to augment Akkermansia abundance in gut microbiota for improved PD-1 blockade efficacy in tumor suppression**

*Shuangying Qiao^1, 2, 3 #^, Liu Yang^1, 2, 3 #^, Haibang Hao^4 #^, Qiuxia Ding^4, 5, #^, Feng Ding^2, 3 #^, Zheng Chen^1, 2 #^, Jinfang Zhang^1^, Yun He^1,2^, Meng Li^3^, Jun Xu^1^, Chao Wang^6^, Aiping Lu^1, 2, 3^ *, Fangfei Li^1, 2, 3^ **


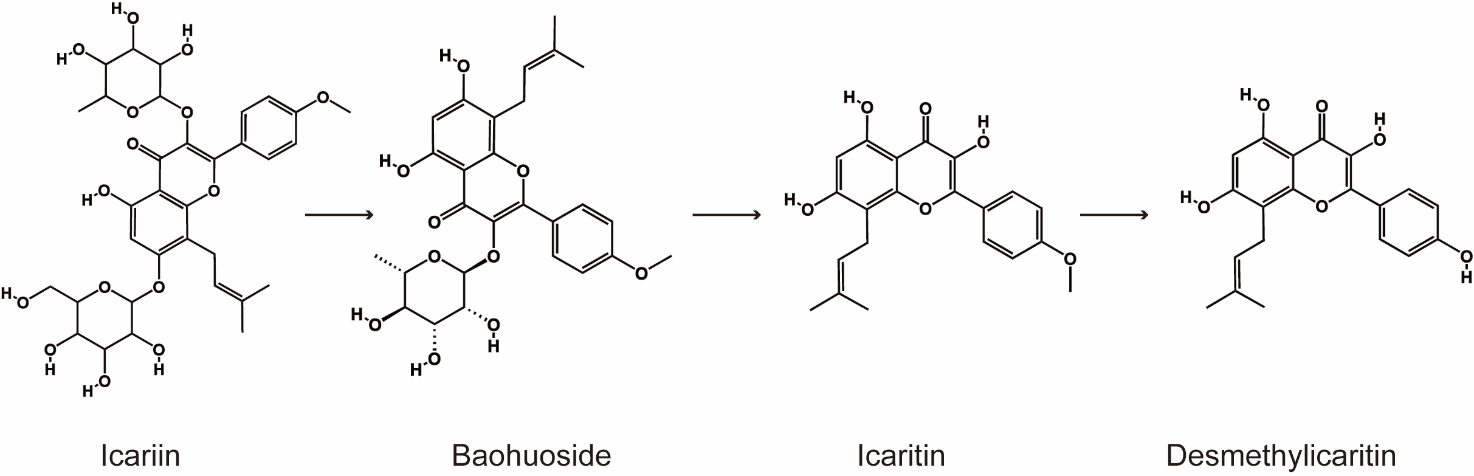


**Figure S1.** Structure of icariin and its metabolites (Baohuoside, Icaritin, Desmethylicaritin).


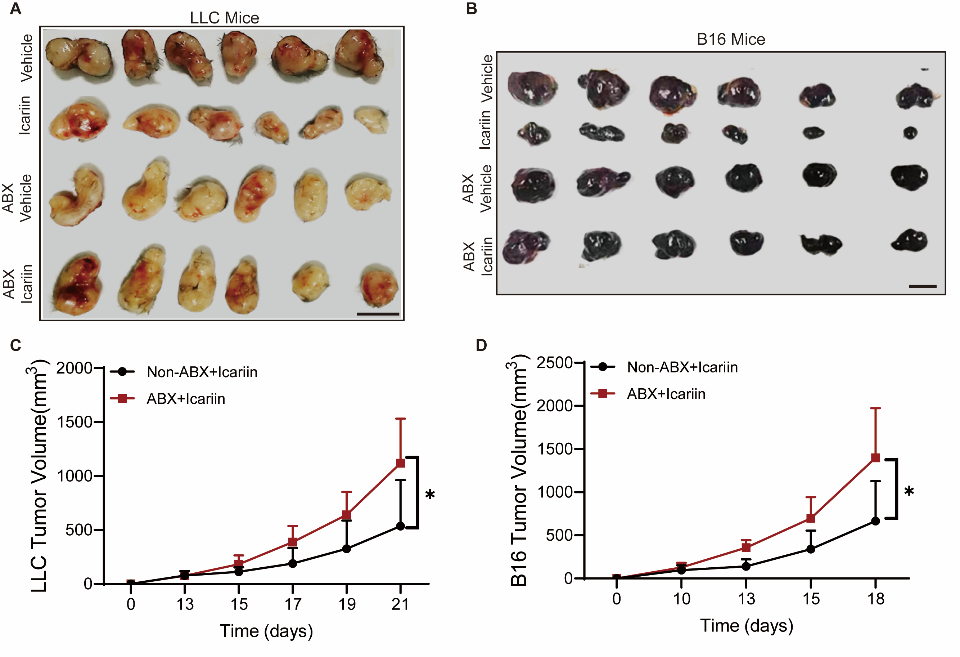


**Figure S2. Gut microbiota mediates icariin's antitumor efficacy in LLC and B16 mouse models. A.** Tumor photographs of non-ABX and ABX LLC tumor-bearing mice treated with icariin (70mg/kg), vehicle, respectively (n=6/group), scale bar: 1cm. **B.** Tumor photographs of non-ABX and ABX B16 tumor-bearing mice treated with icariin (70mg/kg), vehicle, respectively (n=6/group), scale bar: 1cm. **C.** Tumor volumes in LLC tumor-bearing mice treated with icariin (70 mg/kg), with or without ABX treatment (n = 6 per group), scale bar: 1cm. **D.** Tumor volumes in B16 tumor-bearing mice treated with icariin (70 mg/kg), with or without ABX treatment (n = 6 per group).


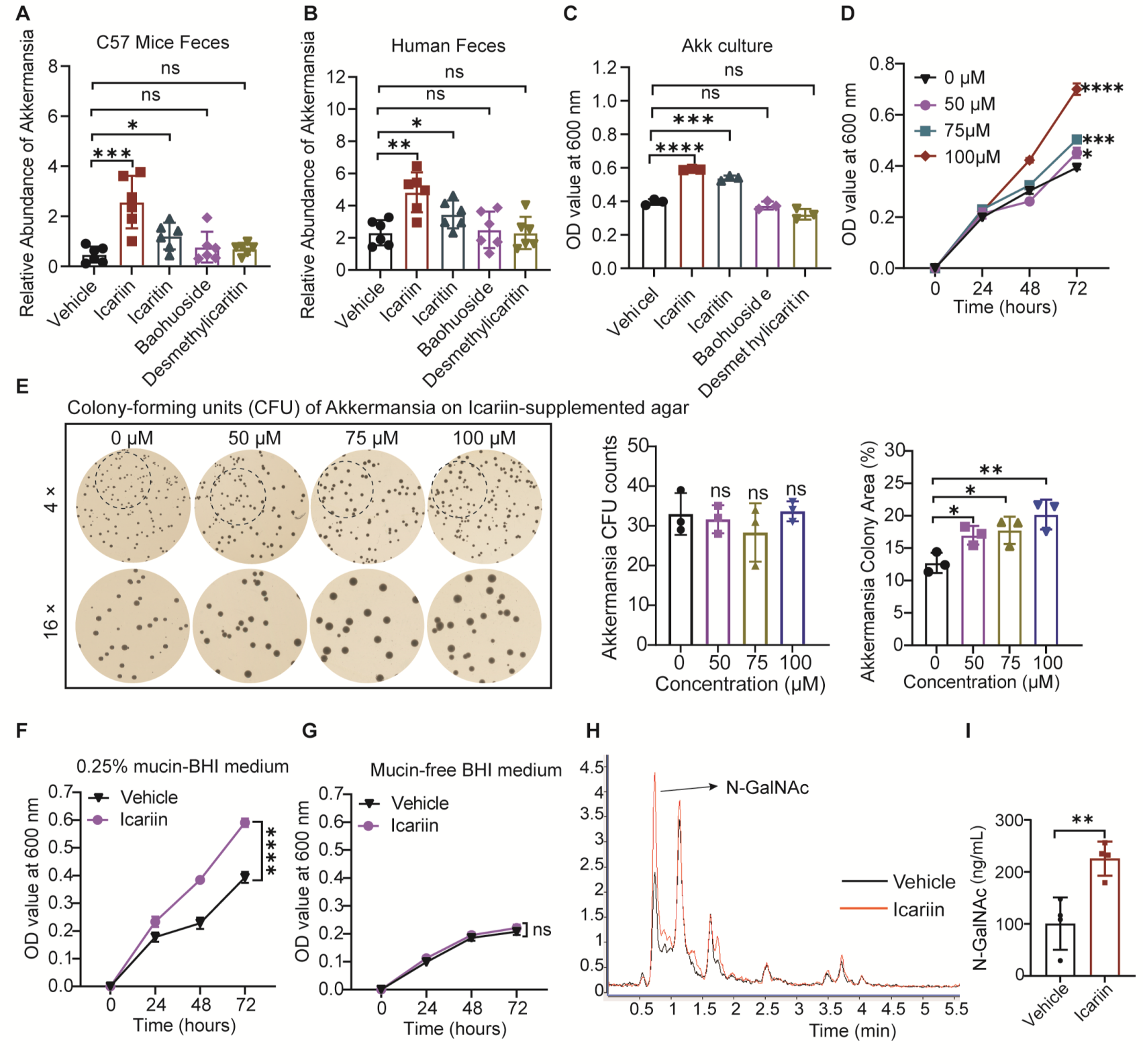


**
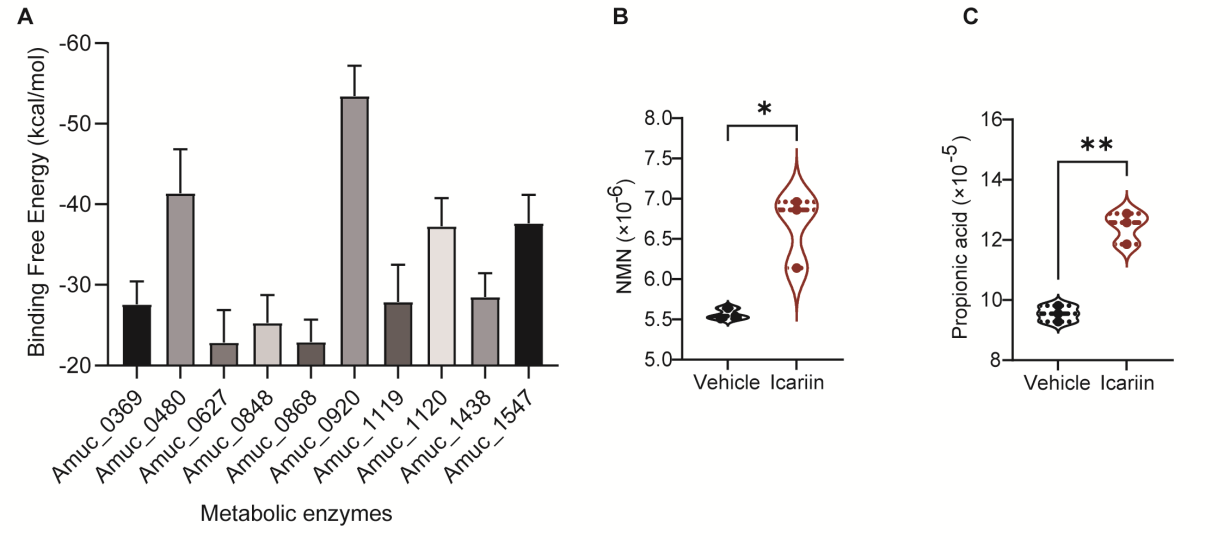
Figure S3.** The statistical analysis of Akk colony-forming units (CFU) cultured on icariin-supplemented 0.25% mucin-BHI agar plates (0–100 µM), with 16× magnified images corresponding to the dashed circles in the 4× images.

**Figure S4. Icariin enhanced the activity of N-acetylgalactosaminidase Amuc_0920 by stabilizing key residues at the binding sites for the substrate GalNAc. A.** The barplot of the changes in binding free energy between the ten enzyme proteins and Icariin over the interval of 100 simulation frames. **B.** Metabolomic analysis of NMN levels in Akk cultured in 0.25% mucin-BHI medium with eother vehicle or 100 μM icariin. Two-tailed unpaired t-test: *p < 0.05; **p < 0.01. ***p<0.001.


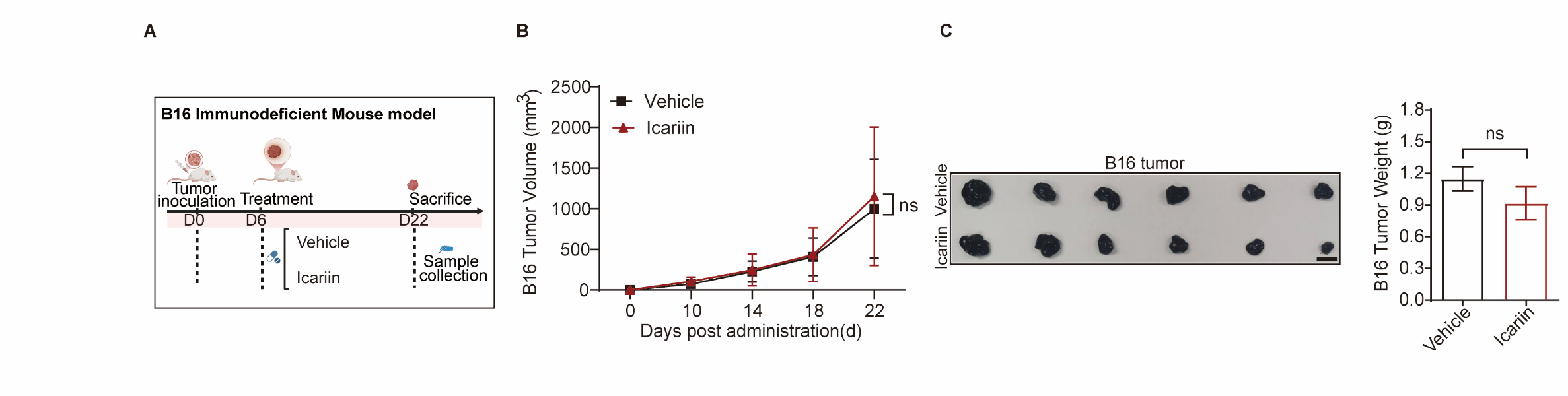


**Figure S5. Icariin's antitumor efficacy significantly declines in B16 immunodeficient mouse model despite increasing Akk abundance. A.** Experimental design of icariin treatment in B16 SCID-NOD mouse model. **B.** Tumor growth curve in B16 tumor-bearing SCID-NOD mice treated with icariin (70mg/kg) or vehicle (n=6/group). **C.** Tumor photographs and statistics of LLC tumor-bearing SCID-NOD mice treated with icariin or vehicle (n=6/group), scale bar: 1cm. Two-tailed unpaired t-test: *p < 0.05, **p < 0.01.


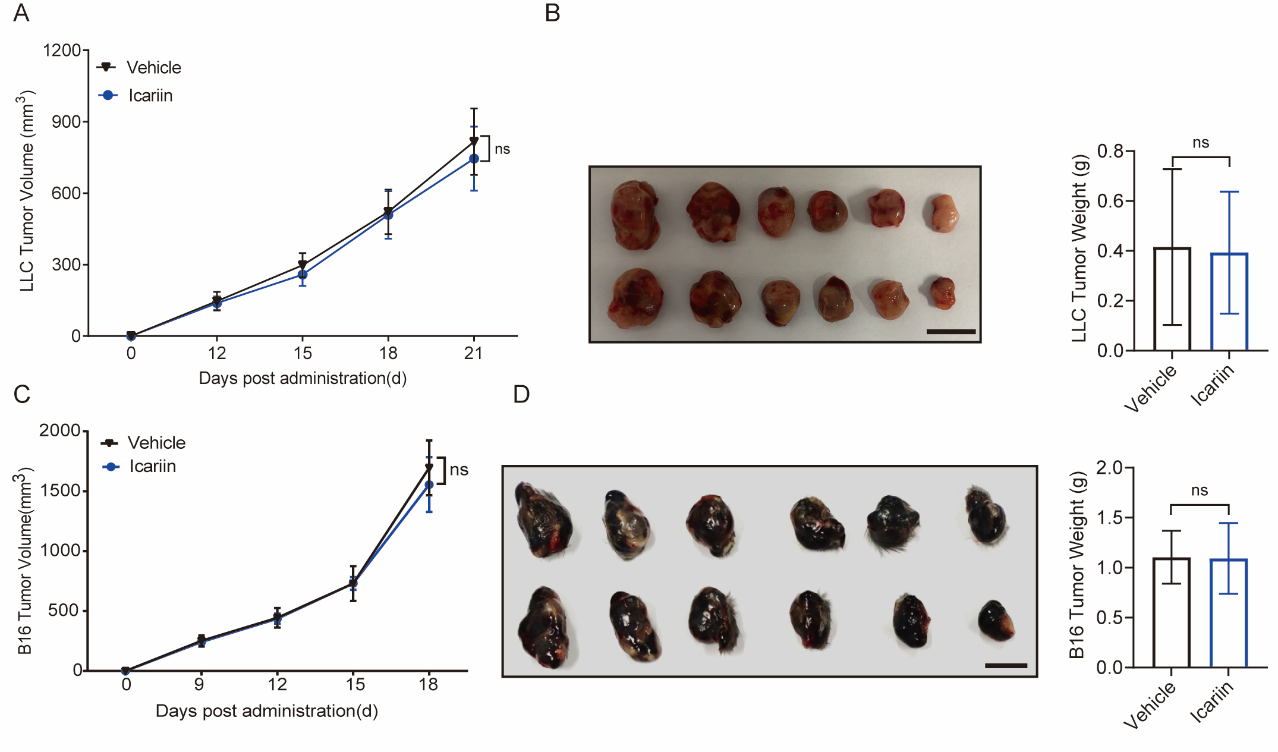


**Figure S6. The antitumor efficacy of icariin is significantly reduced in LLC and B16 tumor-bearing C57BL/6 background Rag1-KO immunodeficient mouse models (B6;129S-Rag1^tm1(loxP-EGFP-PolyA-loxP-Neo-loxP)^Smoc). A.** Tumor growth curve in LLC tumor-bearing C57BL/6 background Rag1-KO immunodeficient mice treated with icariin (70mg/kg) or vehicle (n=6/group). **B.** Tumor photographs and statistics of LLC tumor-bearing C57BL/6 background Rag1-KO immunodeficient mice treated with icariin or vehicle (n=6/group), scale bar: 1cm. **C.** Tumor growth curve in B16 tumor-bearing C57BL/6 background Rag1-KO immunodeficient mice treated with icariin (70mg/kg) or vehicle (n=6/group). **D.** Tumor photographs and statistics of B16 tumor-bearing C57BL/6 background Rag1-KO immunodeficient mice treated with icariin or vehicle (n=6/group), scale bar: 1cm. Two-tailed unpaired t-test: ns, not significant.


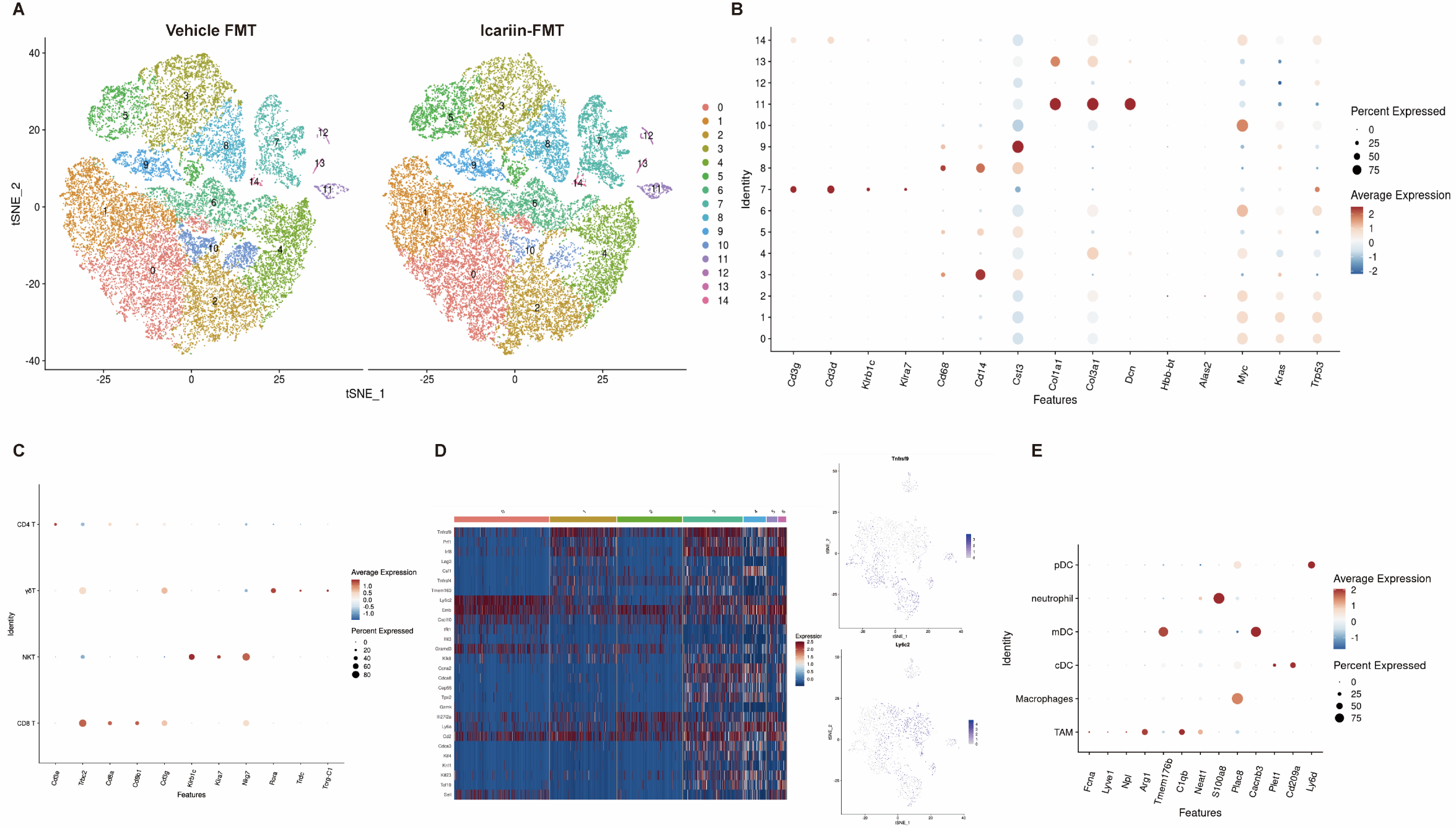

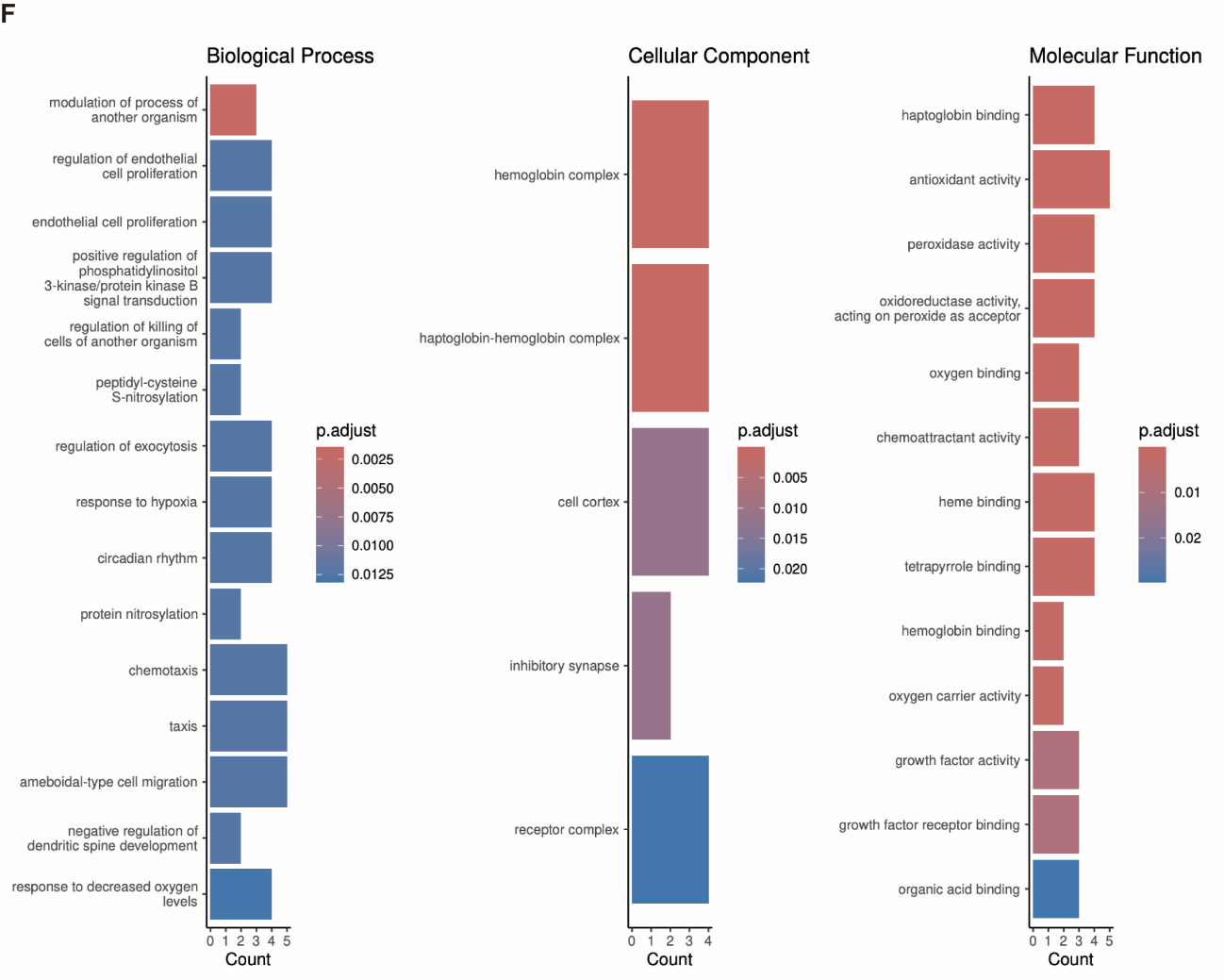


**Figure S7. Marker genes in the clusters of all cell types, and the subtypes of T cell subtypes, CD8 T cell subclusters, and myeloid-derived cells from tumor tissue. A-B.** t-SNE plot **(A)** and FeaturePlot **(B)** indicating the expression of marker genes in the 14 clusters of all cell subtypes. **C.** FeaturePlot indicating the expression of marker genes in the 4 clusters of T cell and NKT cell subtypes. **D.** Heatmap and t-SNE plot demonstrating the representative marker genes in CD8 T cell subclusters. **E.** FeaturePlot indicating the expression of marker genes in the 6 clusters of Myeloid cell subtypes. **F**. The Gene Ontology (GO) of Differential Expression Gene (DEG) from Malignant cells between vehicle FMT group and icariin FMT groups.


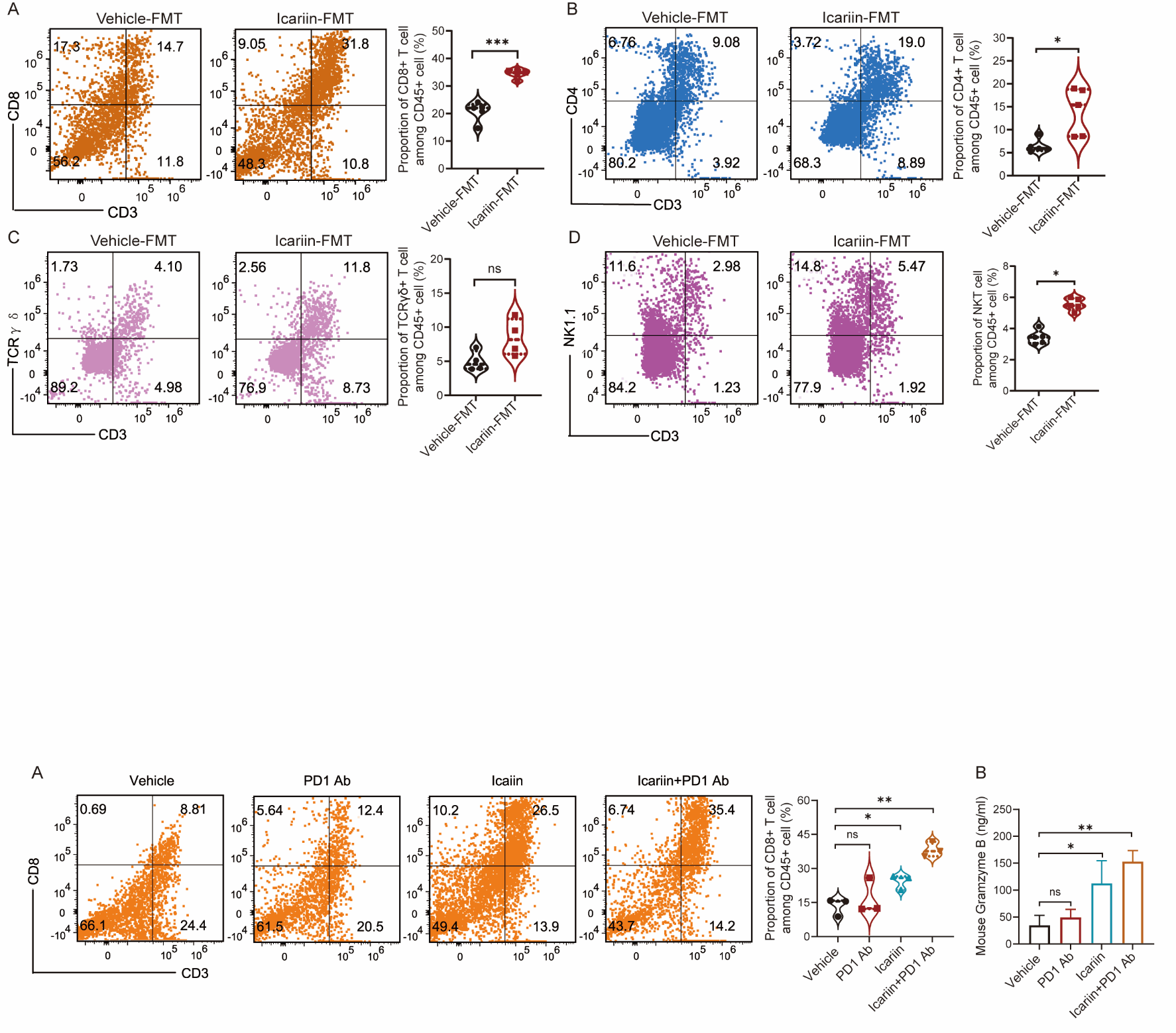


**Figure S8. Icariin-FMT treatment remodeled the intratumoral immune landscape in LLC tumor-bearing mice. A.** Proportion and statistical analysis of CD8+ T cell in tumor tissues of LLC tumor-bearing mice treated with icariin-FMT or vehicle-FMT (n=5/group). **B.** Proportion and statistical analysis of CD4+T cell in tumor tissue of LLC tumor-bearing mice treated with icariin-FMT or vehicle-FMT (n=5/group). **C**. Proportion and statistical analysis of γδ+ T cell in tumor tissue of LLC tumor-bearing mice treated with icariin-FMT or vehicle-FMT (n=4/group). **D.** Proportion and statistical analysis of NKT cell in tumor tissue of LLC tumor-bearing mice treated with icariin-FMT or vehicle-FMT (n=5/group). Two-tailed unpaired t-test: *p < 0.05, **p < 0.01, ***p < 0.001.


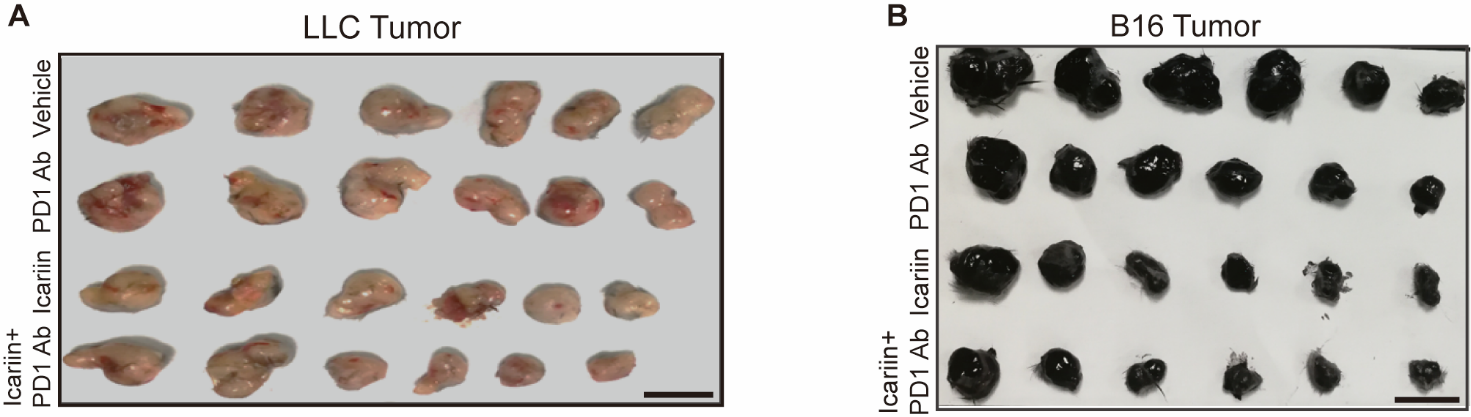


**Figure S9. Icariin enhanced the efficacy of PD-1-based immunotherapy in LLC and B16 mouse models. A.** Tumor photographs of LLC tumor-bearing mice receiving vehicle, anti-PD1 Ab, icariin, or a combination of icariin and anti-PD1 Ab, respectively (n=6/group), scale bar: 1cm. **B.** Tumor photographs of B16 tumor-bearing mice receiving vehicle, anti-PD1 Ab, icariin, or a combination of icariin and anti-PD1 Ab, respectively (n=6/group), scale bar: 1cm.


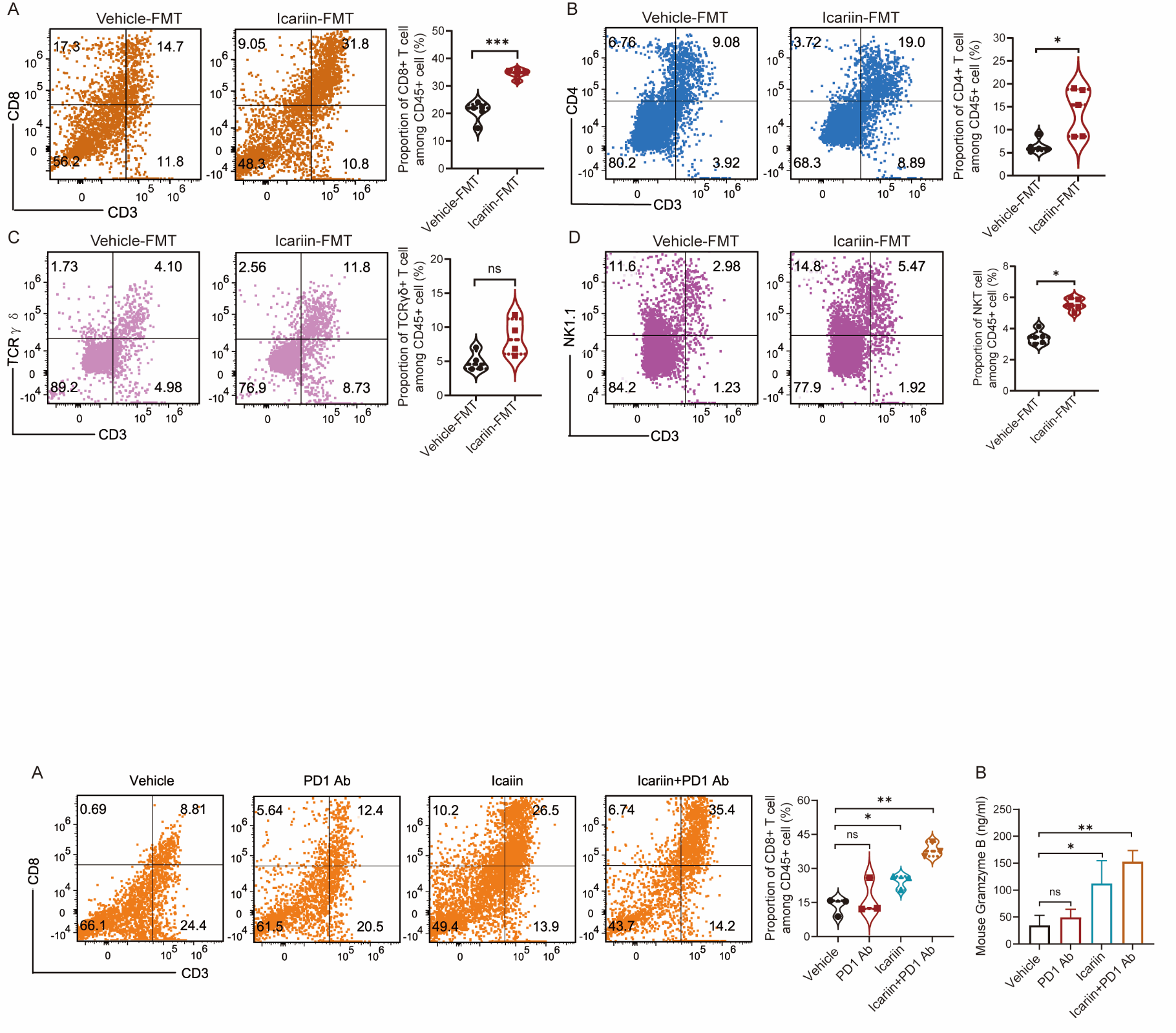


**Figure S10. Icariin enhanced anti PD-1 immunotherapy efficacy via increased intratumoral CD8+ T cell infiltration. A.** Proportion and statistical analysis of CD8+ T cell in tumor tissue of LLC tumor-bearing mice receiving vehicle, anti-PD1 Ab, icariin, or a combination of icariin and anti-PD1 Ab, respectively (n=3/group). **B.** The levels of mouse Granzyme B in tumor tissues were measured in LLC tumor-bearing mice treated with vehicle, anti-PD1 Ab, icariin, or a combination of icariin and anti-PD1 Ab (n=3/group). Two-tailed unpaired t-test: *p < 0.05, **p < 0.01.


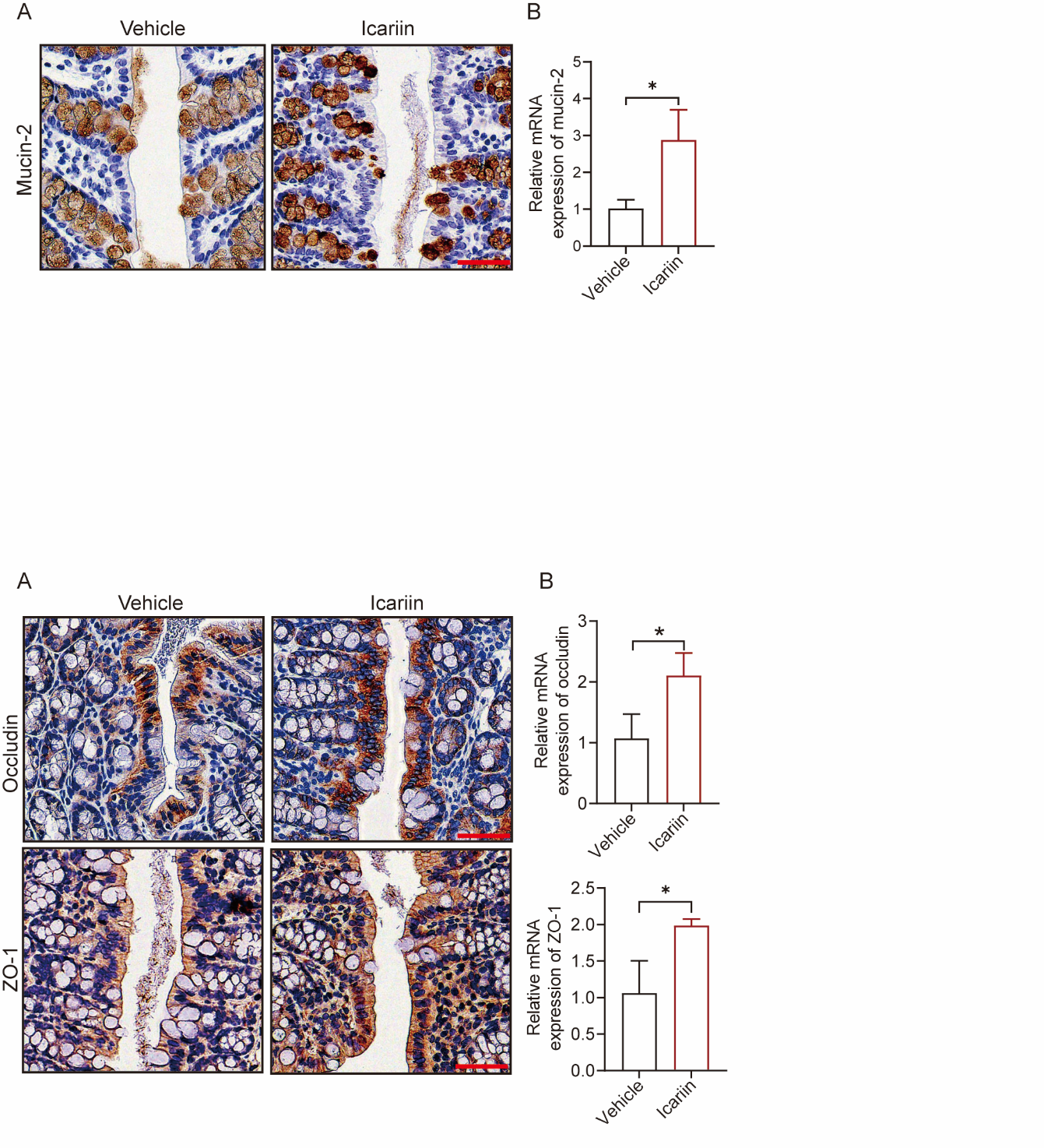


**
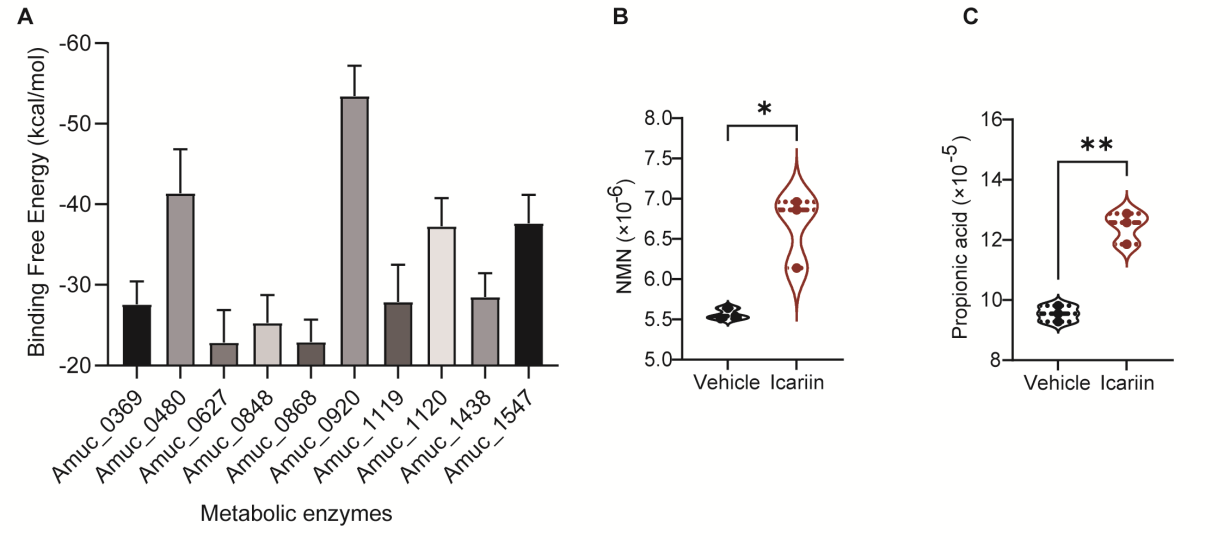
Figure S11.** **Icariin promoted mucin-2 expression in the colon tissues of LLC tumor-bearing mice. A.** Immunohistochemical (IHC) analysis of Mucin-2 expression in colon tissues from LLC tumor-bearing mice treated with icariin (70 mg/kg) or vehicle, scale bar: 50μm. **B.** qPCR analysis of Mucin-2 mRNA expression levels in colon tissues from LLC tumor-bearing mice treated with icariin (70 mg/kg) or vehicle. Two-tailed unpaired t-test: *p < 0.05.

**Figure S12.** Metabolomic analysis of propionic acid levels in Akk cultured in 0.25% mucin-BHI medium with either vehicle or 100 μM icariin. Two-tailed unpaired t-test: *p < 0.05, **p < 0.01.


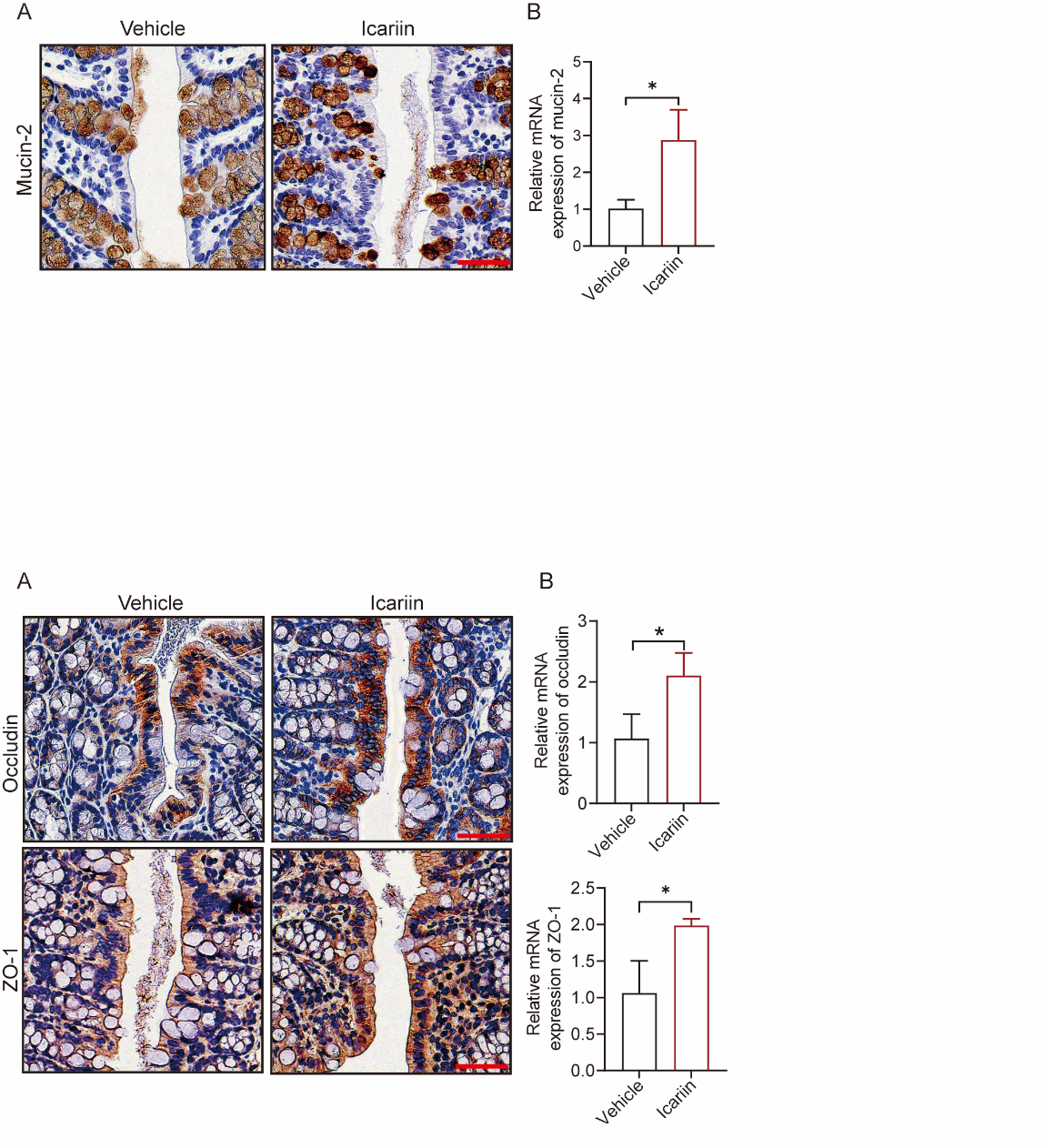


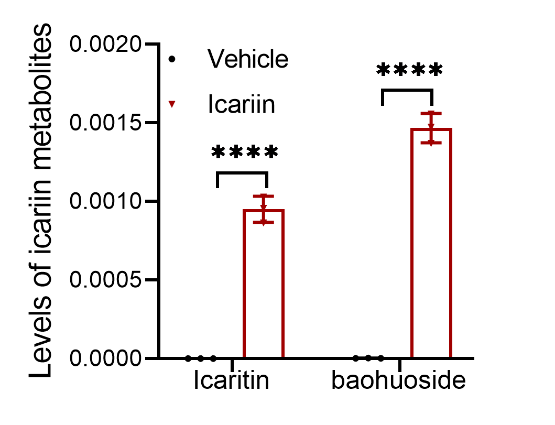
**Figure S13. Icariin promoted intestinal barrier function proteins ZO-1 and Occludin expression in the colon tissues of LLC tumor-bearing mice. A.** Immunohistochemical (IHC) staining illustrating the intestinal barrier function proteins ZO-1 and Occludin expression in colon tissues from LLC tumor-bearing mice treated with icariin (70mg/kg) or vehicle, scale bar: 50μm. **B.** qPCR analysis of ZO-1 and Occludin mRNA expression levels in colon tissues from LLC tumor-bearing mice treated with icariin (70 mg/kg) or vehicle. Two-tailed unpaired t-test: *p < 0.05.

**Figure S14.** Metabolic analysis of icariin metabolites levels in Akk cultured in 0.25% mucin-BHI medium supplemented with either vehicle or 100 μM icariin *in vitro*. Two-tailed unpaired t-test: *p < 0.05; **p < 0.01, ***p<0.001, ****p<0.0001.


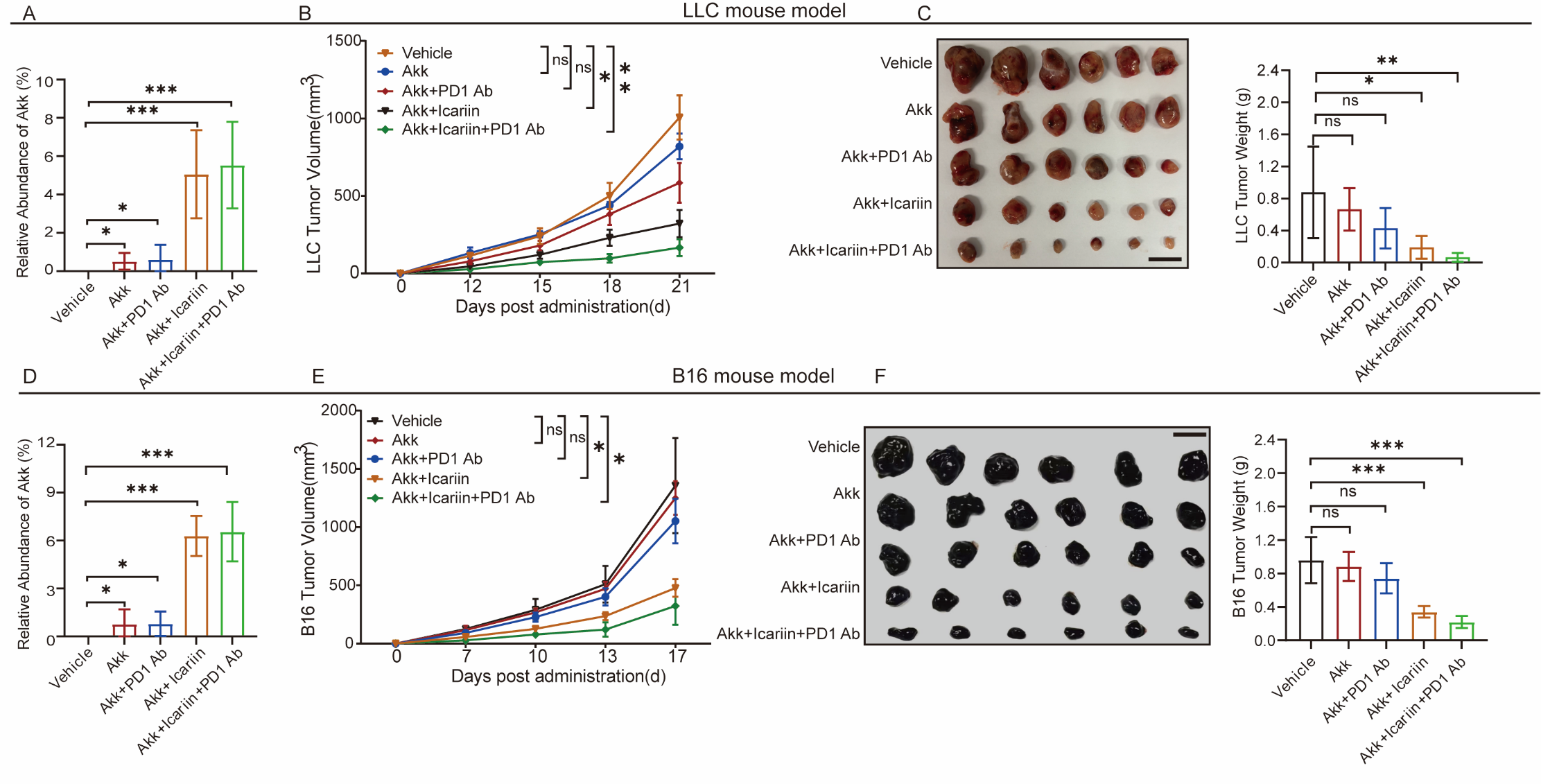


**Figure S15. The combination of icariin and Akk supplementation promoted Akk colonization and significantly enhanced the anti-tumor activity of PD-1 blockade in both LLC and B16 mouse models with lacking Akk. A.** The relative Abundance of fecal Akk in LLC tumor-bearing mice treated with Vehicle, Akk, Akk +PD1 Ab, Akk+Icariin, Akk+Icariin+PD1 Ab (n=6/group) after treatment. **B.** Tumor growth curve in LLC tumor-bearing mice treated with Vehicle, Akk, Akk +PD1 Ab, Akk+Icariin, or Akk+Icariin+PD1 Ab (n=6/group) after treatment. **C.** Tumor photographs and statistical analysis of tumor weight in LLC tumor-bearing mice Vehicle, Akk, Akk +PD1 Ab, Akk+Icariin, or Akk+Icariin+PD1 Ab (n=6/group) after treatment, scale bar: 1cm. **D.** The relative Abundance of fecal Akk in B16 tumor-bearing mice treated with Vehicle, Akk, Akk +PD1 Ab, Akk+Icariin, Akk+Icariin+PD1 Ab (n=6/group) after treatment. **E.** Tumor growth curve in B16 tumor-bearing mice treated with Vehicle, Akk, Akk +PD1 Ab, Akk+Icariin, or Akk+Icariin+PD1 Ab (n=6/group) after treatment. **F.** Tumor photographs and statistical analysis of tumor weight in B16 tumor-bearing mice Vehicle, Akk, Akk +PD1 Ab, Akk+Icariin, or Akk+Icariin+PD1 Ab (n=6/group) after treatment, scale bar: 1cm. Two-tailed unpaired t-test: *p < 0.05, **p < 0.01, ***p < 0.01.
